# Microplastics in aquatic systems: A comprehensive review of its distribution, environmental interactions, and health risks

**DOI:** 10.1007/s11356-024-35741-1

**Published:** 2024-12-13

**Authors:** Divya Pal, Roshan Prabhakar, Visva Bharati Barua, Ivar Zekker, Juris Burlakovs, Andrejs Krauklis, William Hogland, Zane Vincevica-Gaile

**Affiliations:** 1 Department of Biology and Environmental Science, Linnaeus University, SE-392 31 Kalmar, Sweden; 2 Department of Ecology Environment & Plant Sciences (DEEP), Stockholm University, Stockholm, Sweden; 3 Department of Materials and Environmental Chemistry (MMK), Stockholm University, Stockholm, Sweden; 4 Department of Civil and Environmental Engineering, University of North Carolina at Charlotte, 9201 University City Boulevard, Charlotte, NC 28223 USA; 5 Institute of Chemistry, University of Tartu, 14a Ravila St, Tartu, Estonia; 6 Riga Technical University, Riga, Latvia; 7 Norwegian University of Science and Technology (NTNU), Trondheim, Norway; 8 Environmental Engineering and Recovery, Department of Biology and Environmental Science, Faculty of Health and Life Sciences, Linnaeus University, SE-392 31 Kalmar, Sweden; 9 Department of Environmental Science, University of Latvia, Jeglavas Street 1, Riga, LV-1004 Latvia

**Keywords:** Aquatic environment, Bioaccumulation, Health risk, Quantification, Toxicity

## Abstract

**Abstract:**

Microplastics (MPs) have become a critical pollutant, accumulating in aquatic ecosystems and posing significant environmental and human health risks. Approximately 5.25 trillion plastic particles float in global oceans, releasing up to 23,600 metric tonnes of dissolved organic carbon annually, which disrupts microbial dynamics. MPs arise from the breakdown of larger plastics, degraded by photodegradation, thermal degradation, and biological processes, which are influenced by polymer type and environmental factors. As carriers, MPs absorb and transport contaminants such as heavy metals, per- and polyfluoroalkyl substances (PFAS), and persistent organic pollutants (POPs) across trophic levels, thereby increasing toxicity within food webs. Key aquatic organisms, including microalgae, molluscs, and fish, experience cellular toxicity, oxidative stress, and disruptions in essential functions due to MP ingestion or adhesion, raising concerns about their bioaccumulation in humans through ingestion, inhalation, and dermal contact. The complex surface chemistry of MPs enhances their pollutant adsorption, a process modulated by environmental pH, salinity, and contamination levels, while aging and structural attributes further impact their bioavailability and toxicity. This review consolidates knowledge on MPs’ occurrence, transformation, pollutant interactions, and methodologies for sampling and analysis, emphasizing advancements in spectroscopy and imaging techniques to improve MP detection in aquatic environments. These insights underscore the pressing need for standardized analytical protocols and comprehensive toxicological research to fully understand MPs’ effects on ecosystems and human health, informing future mitigation strategies and policy development.

**Graphical abstract:**

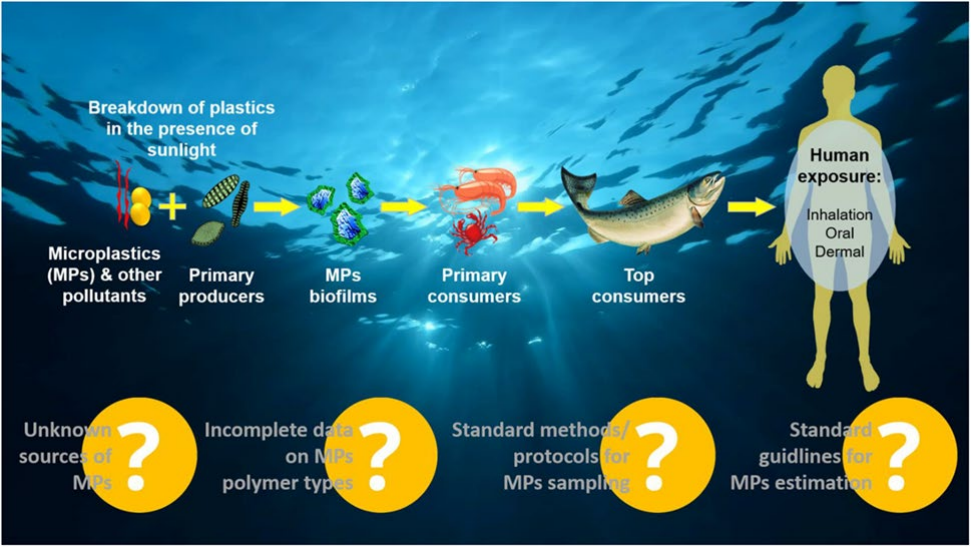

**Supplementary Information:**

The online version contains supplementary material available at 10.1007/s11356-024-35741-1.

## Introduction

Global demand for plastics has surged over the past seven decades, resulting in widespread plastic prevalence across modern environmental systems (Campanale et al. 2020). This exponential augmentation in plastic production has contributed to a rise in plastic waste deposition across diverse environments (Ebrahimi et al. 2022; Spreafico and Russo 2023) (Table 1) (Fig. S1). According to studies, there are approximately 5.25 trillion bits of plastic floating on the ocean’s surface, although their impact on trophic chains remains unclear (Chaturvedi et al. 2020). Plastics release up to 23,600 metric tonnes of dissolved organic carbon annually, of which 60% becomes readily available to microbial populations within 5 days (Romera-Castillo et al. 2018). Inadequate handling practices lead plastics to follow diverse pathways in the environment, contributing to significant environmental challenges (Geyer et al. 2017; Zhang et al. 2021a, 2021b). Particular concern surrounds particles with sizes < 5 mm and 0.1 μm due to their longevity, small size, increased surface-to-volume ratio, and their potential to penetrate living cells, eliciting adverse effects (Cole et al. 2013; Peixoto et al. 2019). Commonly found plastic types in the environment include polypropylene (PP), polystyrene (PS), polyvinyl chloride (PVC), polyethylene terephthalate (PET), and polyethylene (PE) (Güven et al. 2017) (Table 1).

**Table 1:** Recent studies reported the concentration of MPs in different seas

Study area	Dominant debris type	Polymers	Concentration (particles/kg DW)	Explanations	References
Andaman Sea	Fibers	Acrylic, PE, Nylon, and PEP	15.36	Increased anthropogenic activities and uncontrolled plastic disposal along the coastline, semi-treated/non-treated urban effluent discharge increased the pressure of MPs Amplified shipping and fishing activities triggered the fibrous MPs in the Andaman region	Goswami et al. (2021)
Andaman Sea	Fragment and pellet	Nylon, acrylic, and ionomer	45.17	-	Goswami et al. (2020)
Arabian Sea	Fibers	Acrylic, PE, Nylon, and PEP	128.02	Mixing of low-saline Bay of Bengal water into the Arabian Sea that could transport floating MPs from the coastal areas Abundance of laundry wastes with a great amount of acrylic fabric and increased fishing and shipping industry that added the large number of synthetic fibers in the Arabic Sea	Goswami et al. (2021)
Arabian Sea (Coast)	Fragmented	PE and PP	664	Increased industrial and urban activities near the coastline Mechanical and oxidative weathering of large plastic particles	Yaranal et al. (2021)
Arctic Sea	Fragment	PE, PP, and PA	42–6595	Thermohaline circulation of MP debris from Northern Europe to the North and melting of sea ice released the MPs in the Arctic Sea Excessive use of PP and PE in packaging materials and fishing gears increased their amount in the Europe region	Bergmann et al. (2017)
Baltic Sea	Fibers	-	34	Mixing of discharge released from wastewater	Zobkov and Esiukova (2017)
Baltic Sea	Fibrous	PE, PP, PET, PDMS, PVC	863	The oceanographic factors like sediment fraction, surface wave current, and water column structure (i.e., density of MPs jumps at the thermocline and pycnocline levels) are responsible for mixing of MPs Fiber distribution in bottom deposits depends on erosion/transition/accumulation zones	Chubarenko et al. (2022)
North Sea	Fibers		65.95	The geometry of this location regardless of any other area is very unique. Here, the flushing rate is comparatively very low and the narrow entrance can cause the incidence of tidal eddies. The MP floating into such areas gets trapped in the vortex and settles down on the bottom of the seafloor	Claessens et al. (2011)
Black Sea	Fibers	PE and PP	106.7	The storms and wind imply the surface mixing and redistribution of MPs in the water column and sea bed Occurrence of polyamide fibers is related to maritime usages, such as fishing activities	Cincinelli et al. (2021)
Black Sea	Fibers	PE and PP	98	Fluvial transport of MPs from poorly treated wastewater or inadequately treated sewage sludge in Black sea	Pojar et al. (2021)
Bohai Sea	Fibers	PEVA, LDPE, and PS	102.9–163.3	The semi-closed geographical structure surrounded by densely packed industrial and urban structures is responsible for MPs load in this sea	Yu et al. (2016)
Bohai Sea	Fibers and fragments	Rayon, PET, CP, PA, PE, PP, and PC	137	The weak hydrodynamic force favors the settling of fine particulate matter and fine sediment, which is suitable for the deposition of small-sized MPs in the bottom of the sea	Zhang et al. (2022)
Caribbean Sea	Fibers	-	261	The tourism and population near the shoreline promoted the MP pollution	Bosker et al. (2018)
Caribbean Sea	Fragments	-	1109	Flourished human activities along the coastal line, especially the generation of high amounts of single-use plastics.	Rangel-Buitrago et al. (2021)
Caspian Sea	Fibers	PS and PET	196.67	Influx of rivers, Gorganrud, Nokandeh, and Qarasu, promotes the entry of MPs into the sea The anti-clockwise circulation of water from the westerns part to the eastern of the southern Caspian Sea is responsible for transfer of MPs	Manbohi et al. (2021b)
Chukchi Sea	Fibers	PP, PET, and rayon	31.6	In the course of melting process entrained in Arctic Sea ice, released MPs enhanced their number in the Chukchi Sea	Mu et al. (2019)
Da Nang Coast	Synthetic fiber	PA, PVOH, Polyester, PET, PAN, and PAK	9283	Man-made activities such as the discharge of industrial wastewater, solid waste, and landfill leachate have increased the MPs number in this area	Tran Nguyen et al. (2020)
Eastern Baltic Sea	Fibers and fragments	-	490	The discharge and buoyant particles from various basins accumulate in this region Secondly, the cyclonic current arrangement of this area promotes the recirculation of surface water in the basin for the mean cyclonic current structure of the BP recirculates/traps the surface water in the basin for a lengthier period of time, which supports the settlement of MPs on the sea floor	Mishra et al. (2022)
Great Australian Bight	Fragments	Polyisoprene (rubber/latex), PU, polyester, and PP	13.6	The seafloor slope angle and MPs abundance are correlated with each, i.e., area having a steep slope seafloor angle also having a high MP number and *vice-versa*	Barrett et al. (2020)
North Yellow Sea (Qingduizi Bay)	Fibers	PET and CP	33.15	Geographically, this area is semi-closed; thus, an exchange of external water is relatively low that favored the accumulation of MPs	Chen et al. (2022a)
Red Sea	Fragments	PE	1–160	The amplified number of MPs in the Red Sea is either linked with industrial areas or densely populated zones. Lack of recycling infrastructure also promotes the growth of MPs here	Ruiz-Compean et al. (2017)
Red Sea	Fibers and granules	PP and HDPE	nd–119	The intense solar radiation and high temperature of Saudi coastal area enhance the degradation of plastic materials into micro/nanosized plastic materials	Al-Lihaibi et al. (2019)
South Africa Coasts	Fibers	-	80–87	Especially, the mixing of Mzimvubu and Tugela rivers into the South African coasts has elevated the microfiber load in sediment, because most of the part of river basins suffered from the influx of wastewater treatment plant discharge	De Villiers (2018)
Southern Baltic Sea	Fibers and fragments	PP, PE, PS	76–295	Population density and coastal development are the major sources of MPs	Urban-Malinga et al. (2020)
Southern Black Sea	Fragments	PE and PET	181–944	The distribution of MPs in the Southern Black Sea is governed by the interactions between wind and oceanographic topographies such as fronts and eddies that craft an area for MP deposition	Eryaşar et al. (2021)
Southern Caspian Sea	Films and fibers	PE, PP, and PET	246	Influx of local rivers and hydrodynamics along with activities like fishing and industrial discharge are the predominant sources of MPs in the Southern Caspian Sea	Manbohi et al. (2021a)
South China Sea	Granular	Polyester, Rayon, and Nylon	7705	The influence of river discharge and coastal activities is the major source of MPs in this area	Cui et al. (2022)
Southern North Sea	Spheres and fibers	PP, acrylates, PU, and PA	2.8–1188.8	The fine sediment texture of North Sea is responsible for the abundance of MPs. Moreover, incorporation of buoyant MPs into clusters can be another reason for occurrence of MPs the benthic boundary layer	Lorenz et al. (2019)
Tokyo Bay	Fibers and beads	PEP, PE, PAK, PP, PVC, and PCL	1845–5385	Because of the input of high levels of MPs into the canal and the degree of their sedimentation is relatively very high (2–3.5 cm/year) that stocks the MPs in Tokyo Bay	Matsuguma et al. (2017)
Tyrrhenian Sea	Filaments, fragments, and films	Nylon, PU, PE, and PET	1.70	The Tyrrhenian Sea is well known for its high maritime traffic because this Sea provides a route for connection between Elba island and mainland. The load of MPs along the shipping routes is very high because of the use of epoxies, nylon ropes, nets, etc., in ferries that strike the load of MPs here	Mistri et al. (2020)
Indian Coastline	Fibers and fragments	PE, PP, and PET	12.22–439	Because the huge production of solid waste (around 62MT, collected ~82%) in India, of which only 28% can be treated and the rest is dumped in open areas is the major source of MPs Furthermore, unique oceanic dynamics and monsoon patterns can be another reason for high level of MPs on the Indian Coastline	Ranjani et al. (2021)

*PP* polypropylene, *PE* polyethylene, *PS* polystyrene, *PET* polyethylene terephthalate, *PDMS* polydimethylsiloxane, *PVC* polyvinyl chloride, *CP* cellophane, *PA* polyamide, *PC* polycarbonate, *PEP* polyethylene-polypropylene co-polymers, *PU* polyurethane, *PVOH* poly-ethylene vinyl alcohol copolymers, *PAN* polyacrylonitrile, *PAK* polyacrylate, *PCL* polycaprolactone, *PEVA* polyethylene vinyl acetate, *LDPE* light density polyethylene, *MT* metric tonnes, *PMMA* polymethylmethacrylate

Under various environmental pressures, such as physical, chemical, or biological influences, plastic materials degrade into smaller particles, forming secondary MPs. Simultaneously, the deliberate inclusion of microscopic plastic particles in industrial processes and household products generates primary MPs in the environment. Based on the size, MPs are categorized as nanoplastics (< 1 μm), microplastics (fragments less than 5 mm in size), mesoplastics (particles measuring between 5 and 25 mm), and macroplastics (pieces exceeding 25 mm in dimension) (Goswami et al. 2020; Chauhan et al. 2021; Pastorino et al. 2021).

MPs are pervasive in various ecological settings, encompassing aquatic, atmospheric, and soil systems (Guo et al. 2020; Liu et al. 2022). Additionally, MPs have been detected in food products, intensifying concerns (Mei et al. 2020). The deterioration of plastics is influenced by their chemical composition and additives, in degradation rates; for instance, a PE bag may take 10–20 years, while a PET bottle can persist for 450–1000 years (Chamas et al. 2020). Everyday items like clothing and cosmetics, composed of polyester and polyamide materials, contribute significantly to MPs in the environment (Napper and Thompson 2016).

Plastics and MPs released into the environment pose a severe threat to aquatic biota (Li et al. 2021). Due to their resemblance to prey, aquatic organisms such as fish, mussels, clams, and others may ingest or absorb MPs, causing physical harm and lethal effects by damaging the digestive system and tissues (Prata et al. 2020). Moreover, MPs facilitate the transport of contaminants in the environment, adsorbing substances such as heavy metals, metalloids, per- and polyfluoroalkyl substances (PFAS), polychlorinated biphenyls (PCBs), and polycyclic aromatic hydrocarbons (PAHs) and releasing them into various environments. This process poses risks to the digestive and respiratory systems of humans and other animals (Reisser et al. 2014; Brennecke et al. 2016; Wu et al. 2021), especially near metal-releasing sites such as antifouling paint and battery industries (Brennecke et al. 2016).

Over the past decade, numerous articles have emerged discussing MPs, encompassing their origins, transport pathways (Wang et al. 2021), and their adsorption ability to environmental contaminants (Wang et al. 2018; Yu et al. 2021) (Fig. S2). Moreover, an increasing number of researchers have sought to explore the interactions between MPs and other environmental contaminants or assessed MPs’ presence in organisms’ tissues. Owing to their diminutive size, MPs are highly susceptible to ingestion by organisms, along with other coupled contaminants. Weber et al. (2021) tested the ecotoxicological effects of PS fragment MPs in freshwater bivalves and concluded that the tested species were unaffected by PS fragment toxicity. Furthermore, in a recent study, a freshwater bivalve species was exposed to environmental PS (rather than laboratory-based samples). The results confirmed that environmental MPs and NPs triggered stronger immune and detoxification responses in the bivalves (Latchere et al. 2023). Nevertheless, it is important to highlight that the mechanisms of action (MoA) and ecotoxicological impacts of contaminants adsorbed to MPs in organisms remain unclear. Thus, elucidating viable pathways for interactions between MPs and various contaminants (organic and inorganic compounds), as well as their MoA in aquatic ecosystems, remains a critical research priority. Additionally, to acquire in-depth knowledge of MPs on the environment, biota, and human beings, it is crucial to analyze and quantify them. Various methods are being employed for sampling and analysis in diverse environmental matrices; nevertheless, there is still no consensus on methodologies tailored to specific environments.

Considering the above facts of MP pollution, the present work focuses on overall aspects of MP pollution starting from its occurrence, behavior, risk, and their characterization analysis. Specific objectives of this work include the following: (1) systematic analysis of factors influencing the genesis of MPs in aquatic systems; (2) investigation of the MoA of various contaminants associated with MPs on aquatic organisms across diverse trophic tiers within the aquatic food web; and (3) discussion of the conceivable risks of MPs on human health; (4) elucidation of effective methodologies for sampling, identification, and quantification of MPs in water and sediment matrices.

**Fig. 1:**
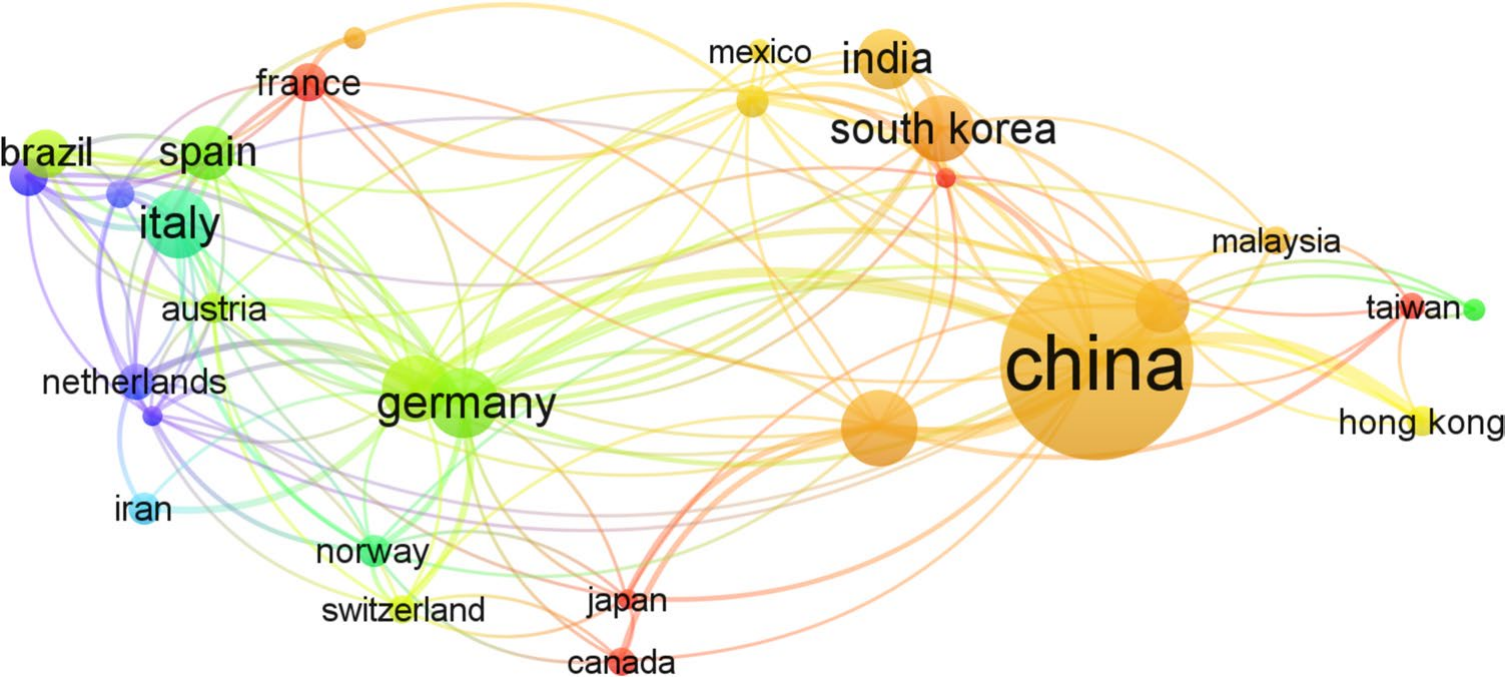
The multinational collaborative network built by co-authorship analyses generated from VOSviewer. The color code represents the clusters and the size of the circle is related to the link strength of all the authors of the specified country. Only the links with a minimum strength of five are displayed

**Fig. 2:**
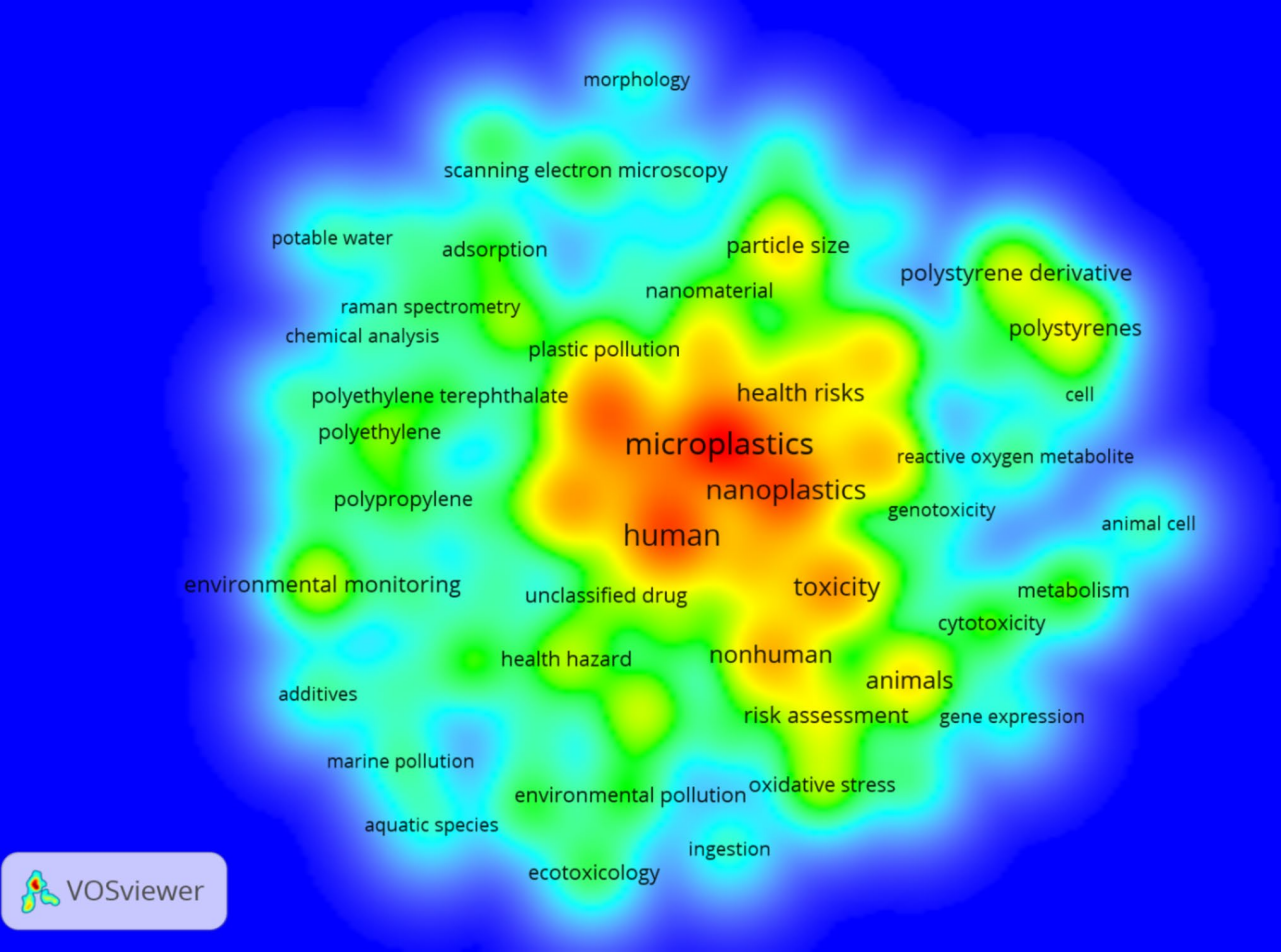
Cluster density analysis of keywords with a word frequency of more than five times. The data come from a literature search with MPs/NPs, ecotoxicity, and human health as the subject terms. The results have been divided into 3 clusters depending on the co-occurrence keywords

The search for relevant publications was conducted through various web resources, utilizing databases like Google Scholar, Web of Science, and Scopus, employing search terms such as “microplastic,” “estimation,” “sediment,” “microalgae,” “contaminant interaction,” and “human health risk.” The search encompassed all available publications until September 2022, revealing only 12 articles on the estimation of microplastics and a single article on the estimation of MPs in sediment (Sundar et al. 2021). Only 17 research articles are listed for human health effects, with the initial study on the impacts of MPs on human well-being published in 2018 by Rist et al. (2018). Additionally, VOSviewer (version 1.6.18) was employed for cluster-based analysis, and Web of Science was used for citation analysis (e.g., network map of countries) (Fig. 1) and co-occurrence (e.g., density map of keywords) (Fig. 2).

## Behavior and transformation of plastics into MPs/NPs in aquatic ecosystems

**Fig. 3:**
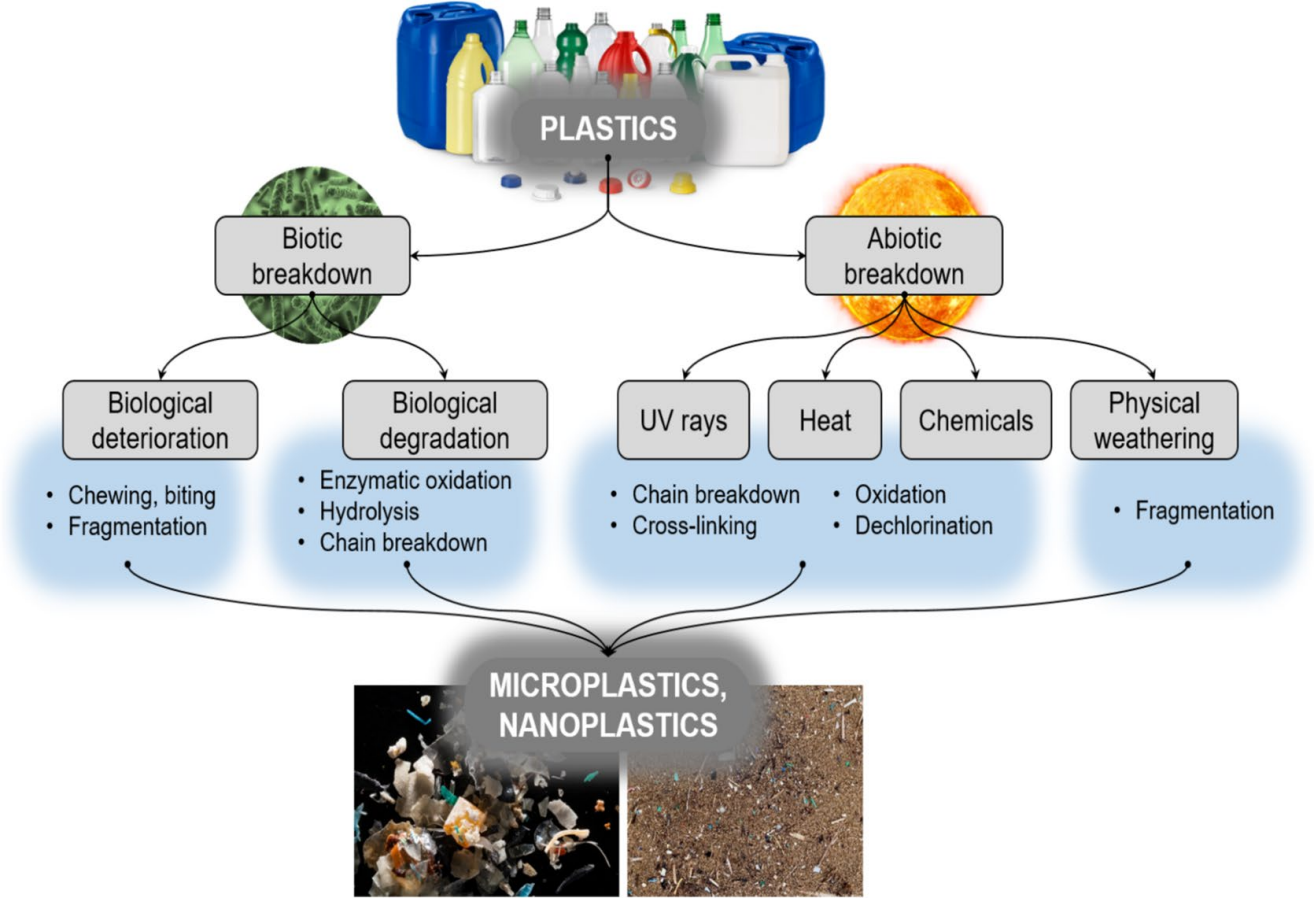
Diagrammatic representation of general processes responsible for the breakdown of plastics in the environment

Plastics are highly resistant to natural degradation, and their persistence in the environment leads to gradual transformation into smaller particles, such as MPs and NPs, which have been detected throughout aquatic ecosystems (Plastic Europe, 2019) (Fig. 3). These transformations result from various abiotic and biotic mechanisms unique to aquatic environments, including photodegradation, thermal and chemical degradation, mechanical fragmentation, and biological processes. Each mechanism varies in effectiveness depending on polymer type and environmental conditions. This section discusses these processes, focusing on their roles, limitations, and comparative effectiveness in aquatic systems.

### Solar breakdown of plastics

Solar UV radiation is one of the primary drivers of plastic degradation in aquatic ecosystems, especially at or near the water surface. UV radiation, particularly high-energy UV-B (290–315 nm) and UV-A (315–400 nm) generates free radicals within plastic polymers, initiating photodegradation that leads to the breakdown of polymer chains (Liu et al. 2019b). This photodegradation process is highly effective for plastics with chromophoric groups that absorb UV light, such as PS and PET. However, many commonly used polymers, including PE and PP, lack natural chromophores, making them more resistant to UV degradation (Fairbrother et al. 2019). Impurities or structural imperfections acquired during manufacturing or environmental exposure can act as chromophores for these polymers, increasing their susceptibility to sunlight-driven breakdown (Law 2017).

Photodegradation rates are influenced by multiple factors, including polymer type, exposure duration, and geographic location, with higher rates observed in tropical and subtropical regions where UV radiation intensity is greater (Kumar et al. 2020). For instance, as carbonyl groups accumulate in PE due to oxidation, they create chromophoric sites that enhance subsequent photo degradability under sustained sunlight exposure, forming smaller organic compounds like aldehydes, ketones, and carboxylic acids (Fairbrother et al. 2019).

However, photodegradation primarily occurs on the water surface, as UV light penetration decreases sharply with depth, especially in turbid or polluted waters (Luo et al. 2022). This limitation reduces photodegradation effectiveness in deeper aquatic zones, making it less impactful for submerged or suspended plastic particles that are shielded from direct sunlight. In comparison to terrestrial environments, where plastics may be exposed to more intense UV exposure, aquatic photodegradation is limited by light availability, emphasizing the importance of other degradation processes in deep water.

### Thermal degradation of plastics in aquatic systems

Thermal degradation, driven by elevated temperatures, plays a key role in the breakdown of plastics, particularly in shallow or coastal aquatic environments exposed to prolonged sunlight. In this process, plastics like PE and PP undergo thermo-oxidative reactions, where heat and oxygen interact to induce radical formation, causing chain scission and, occasionally, cross-linking within the polymer matrix (Zhang et al. 2021a, 2021b). Similar to photodegradation, these reactions lead to the production of hydroperoxides, which subsequently break down into smaller fragments. The combination of UV and thermal energy can have a synergistic effect, accelerating degradation rates in sunlit coastal areas (Pirsaheb et al. 2020).

While elevated temperatures are crucial for thermal degradation, most aquatic environments do not reach the high temperatures required for significant degradation. Typical environmental temperatures in deeper or colder waters fall well below the activation energy needed to trigger thermo-oxidative reactions (Crawford and Quinn 2017; Luo et al. 2022). As a result, thermal degradation is generally limited to surface or shallow areas and is most effective in warmer climates. In contrast, thermal degradation in terrestrial environments, such as in urban or desert areas with extreme temperatures, may occur more rapidly than in aquatic settings. This environmental limitation reduces the overall impact of thermal degradation in oceans and deep freshwater systems, making it only a supplementary factor in plastic breakdown in aquatic systems.

### Chemical breakdown of plastics in aquatic environments

Chemical degradation of plastics in water bodies is influenced by pH, salinity, and the presence of dissolved chemicals and pollutants. Hydrolytic degradation, for instance, is accelerated in aquatic environments with extreme pH levels. Hydrolyzable polymers, like polyamide (PA) and PET, undergo rapid hydrolysis under acidic or basic conditions, leading to the formation of shorter chains and compounds like terephthalic acid and ethylene glycol (Crawford and Quinn 2017). For example, PET in low pH environments degrades faster than in neutral pH, where the hydrolysis rate is significantly slower (Hocker et al. 2014; Zhang et al. 2021a, 2021b).

Salinity also contributes to plastic degradation in marine environments. In high-salinity settings, ionic interactions can weaken the structural integrity of plastics, particularly affecting polymer elongation and tensile strength, which predisposes them to mechanical fragmentation (Zhang et al. 2021a, 2021b). Pollutants like sulfur dioxide (SO₂), nitrogen oxides (NOₓ), and tropospheric ozone (O₃) play a significant role in degradation by generating free radicals that accelerate chain scission in polymer backbones (Weinstein et al. 2016). These pollutants also cause secondary reactions that further facilitate plastic breakdown, particularly in coastal regions where pollutant levels are higher due to proximity to human activity (Liu et al. 2019b).

Despite its relevance, chemical degradation in aquatic environments is typically limited to surface waters where pollutants and UV radiation are most concentrated. In deeper zones, the rate of chemical breakdown slows, resulting in uneven degradation across aquatic environments (Luo et al. 2022). Comparatively, terrestrial environments often exhibit more direct interactions between pollutants and plastics, leading to potentially higher degradation rates in heavily polluted urban areas. This contrast highlights the need for further research on how specific pollutants impact plastic degradation in varied aquatic settings.

### Mechanical breakdown of plastics in aquatic settings

Mechanical fragmentation is an essential process that reduces larger plastic items into MPs and NPs in aquatic environments. Physical forces, such as wave action, sediment abrasion, and particle collisions, contribute significantly to the breakdown of plastics, especially in rivers, shorelines, and estuarine zones (Thakur 2018). Plastics exposed to constant motion in these areas are subject to continuous impact, resulting in fragmentation over time. Mechanical breakdown is especially relevant for polymers with low tensile strength or fracture strain, such as PS and PA, which are more prone to wear from water movement (Salvador Cesa et al. 2017).

Mechanical degradation is highly effective in coastal and riverine areas, where tidal movements and wave energy are intense, creating a consistent source of MPs. However, this process is highly dependent on environmental conditions, such as water currents and sediment load, which vary significantly across different aquatic settings (Wagner et al. 2018). In calm or deep water, where physical forces are reduced, mechanical fragmentation rates decrease, making the process inconsistent across aquatic environments. By comparison, terrestrial environments may experience direct mechanical degradation through processes like trampling or physical abrasion, which can occur more rapidly than in deep aquatic zones (Luo et al. 2022).

### Biological degradation of plastics in aquatic systems

Biological degradation in aquatic ecosystems is facilitated by various microorganisms, including bacteria, fungi, and algae, which colonize plastic surfaces and break down plastics through enzymatic action. Hydrolyzable polymers, such as PET and PA, are more susceptible to microbial degradation, as aquatic microbes produce enzymes like lipase, protease, and esterase that catalyze hydrolysis reactions, reducing plastic polymers into bioavailable smaller molecules (Chen et al. 2020a). Biofilm formation on plastic surfaces in aquatic environments enhances microbial colonization, increasing the degradation rate by providing a stable environment for microbes (Wu et al. 2019b).

Non-hydrolyzable polymers like PE and PP, however, are more resistant to microbial degradation due to their structural stability and absence of functional groups vulnerable to enzymatic attack (Giyahchi and Moghimi 2023). Some microbial communities with lignin-degrading enzymes may facilitate limited degradation of these polymers, but the process is slow and often incomplete in natural settings (Danso et al. 2019; Patrício Silva 2022). In comparison, terrestrial environments, with varied microbial compositions and environmental conditions, may support different degradation rates and efficiencies depending on soil type, pH, and temperature. The slower biological degradation in aquatic settings underlines the need for additional research on microbial communities specific to water environments to optimize degradation processes for non-hydrolyzable plastics.

Thus, the degradation of plastics in aquatic environments varies widely depending on polymer type, environmental exposure, and the primary degradation mechanisms involved. Table 2 summarizes degradation characteristics for commonly found polymers in aquatic systems, highlighting approximate degradation times, primary degradation mechanisms, and major environmental factors influencing their breakdown.

**Table 2:** Degradation characteristics of common polymers in aquatic environments

Polymer type	Degradation time	Degradation mechanism	Major environmental factors	Key notes	References
PE	100 to 1000s of years	Photodegradation, mechanical	UV exposure, biofouling, turbulence	Slow degradation due to lack of chromophores; UV-absorbing impurities enhance breakdown	Fairbrother et al. (2019)
PP	100s to 1000s of years	Photodegradation, thermal	Sunlight, temperature, stress	UV-induced radicals promote degradation; highly hydrophobic, resisting microbial activity	Crawford and Quinn (2017); Law (2017)
PVC	10 to 100s of years	Chemical degradation, thermal	Presence of pollutants (e.g., NO₂, SO₂), pH, salinity	Releases toxic additives; chlorine content contributes to secondary pollution risks	Teuten et al. (2007); Ebrahimi et al. (2022)
PS	50–500 years	Photodegradation, mechanical	UV intensity, wave action, oxygen	UV-sensitive; phenyl rings accelerate photodegradation via free radical formation	Liu et al. (2019b); Kumar et al. (2020)
PET	100–500 years	Photodegradation, hydrolysis	UV exposure, pH, microbial activity	Hydrolyzable; degrades faster in acidic or high-UV conditions; more biodegradable than PE/PP	Zhang et al. (2021a, 2021b)
PA	20–100 years	Hydrolysis, biological	pH, microbial activity, salinity	Amide bonds enhance hydrolysis; microbial enzymes aid degradation under favorable conditions	Danso et al. (2019)
Polylactic acid (PLA)	Months to years (under ideal conditions)	Biological, hydrolysis	Temperature, microbes, moisture	Biodegradable in compost-like conditions; stable in cold aquatic environments	Chen et al. (2022b)

Each of these processes faces specific limitations within aquatic environments, highlighting the complexity and variability of plastic breakdown across different water systems. In comparison to terrestrial environments, where conditions like direct sunlight, temperature fluctuations, and pollutant exposure may enhance degradation, aquatic systems present unique challenges that slow plastic transformation. Future research should focus on understanding how environmental conditions across diverse aquatic settings influence degradation rates, with a particular emphasis on optimizing conditions for the breakdown of persistent plastic types, such as PE and PP, to reduce the longevity and ecological impact of MPs and NPs in marine and freshwater ecosystems.

## Chemical linkage between MPs and organic-inorganic contaminants in the aquatic environment

### Organic pollutants

**Fig. 4:**
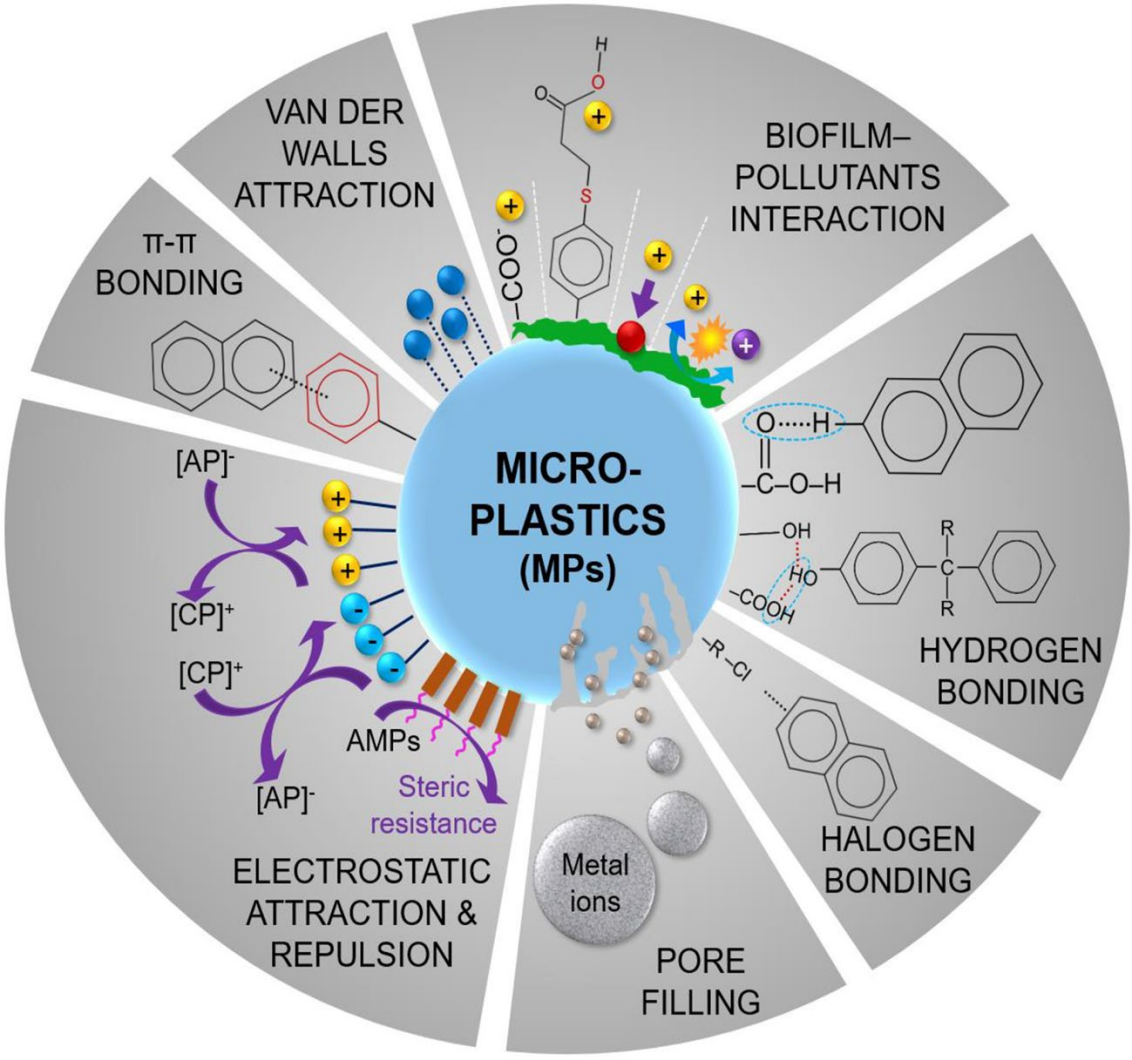
Schematic illustration of possible primary binding mechanisms of organic and inorganic pollutants on MP surface

The primary processes involved in the adsorption of organic pollutants encompass (i) hydrophobic coupling, (ii) partitioning, (iii) electrostatic exchanges, and (iv) non-covalent bonding (Fig. 4) (Guo and Wang 2019; Liu et al. 2022).

#### Hydrophobic coupling between pollutants and MPs

A significant presence of resins in plastics results in pronounced hydrophobicity. Resins are typically categorized into two groups depending on their physical structure: macroporous or macroreticular and highly cross-linked polymers. The primary responsibility for adsorbing organic pollutants lies with MPs carrying these resin types, as they offer substantial surface area and unique functional groups (Chang et al. 2008; Ayman.FM et al. 2023). Because the majority of organic pollutants are hydrophobic and have poor solubility in water, they may readily adsorb on the surface of MPs (Fu et al. 2020). Organic substances with a higher octanol/water partition coefficient (*K*_ow_/*K*_d_) are more prone to adsorb on MPs and *vice versa*. Bisphenol adsorption on the surface of MPs has revealed a significant linear correlation between uptake capacity and hydrophobicity (Log *K*_ow_) (Wu et al. 2019a). Furthermore, the adsorption of perflurooctane sulfonamide (FOSA) on the surface of PE MPs has displayed a strong relationship with Log *K*_ow_ (Liu et al. 2020), highlighting the significance of hydrophobicity in capturing the organic pollutants.

#### Partition effect

The partitioning effect is the dispersion of contaminants between the adjacent aqueous layer surrounding the MPs and the host solution, and it is mostly based on Van der Waals forces following linear adsorption (Liu et al. 2016). The adsorption isotherm study for the uptake of aliphatic and aromatic substances on PE MP surface reveals the ~1 *n* value, specifying the strong linearity and tendency of aromatic substances on MPs through partition coefficient (Hüffer and Hofmann 2016). In one investigation, it was confirmed that the *n* value for Freundlich isotherm in organic drug absorption via PE MPs was equally 1, implying that the isotherm represents linearity (Guo et al. 2019). Thus, the partition coefficient is a crucial factor affecting organic chemical adsorption on MPs.

#### Electrostatic exchange

The electrostatic exchange mechanism is pH-dependent (Guo et al. 2019). When the pH of the operational solution exceeds the zero point charge (pH_zpc_) of the MPs, the surface of the MPs will be saturated with negative ions, which can attract positively charged ions from organic molecules. If the pH of the working solution surpasses the acid dissociation constant (pKa) of the organic compounds, deprotonation occurs, converting the organic substances to anionic form. Conversion of organic compound surface charges from the cation to anion, causing electrostatic repulsion and preventing sorption on the MP surface (Wu et al. 2019a). Thus, the electrostatic exchange process involves the electrification of MPs, ion amount, and ionic state of organic substances.

#### Additional non-covalent bonding

The adsorption process is also influenced by various non-covalent interactions created between organic compounds and MPs, including hydrogen bonding, Van der Walls forces, and π-π bonding, as seen in Fig. 4 (Fu et al. 2021a). The chemical reaction between hydrophilic organic compounds and ionic PA-MPs is primarily governed by hydrogen bonding (Wu et al. 2019a). However, organic materials like DDT and PCBs also bond with MP halogen atoms to generate hydrogen bonds (Wu et al. 2019a). Organic chemicals, such as PAHs and PCBs, are likely to link to PS MPs through π-π bonding (Hüffer and Hofmann 2016). Furthermore, π-π bonding facilitates the binding of other organic compounds to the surface of MPs (Fu et al. 2021a). Van der Walls forces are weak bondings between organic substances and MPs that lack covalent or ionic exchange, mainly occurring between aliphatic polymers.

### Inorganic pollutants (metals) adsorption

The MPs can potentially adsorb heavy metals from the nearby environment and can be a vector of metals to aquatic organisms (Bhagat et al. 2021; Liu et al. 2022). Because of its continual deterioration in the environment, MP surface shape has altered, opening up a variety of routes for heavy metal adsorption (Brennecke et al. 2016). Therefore, it is crucial to expose the mechanisms responsible for heavy metal sorption on MPs (Fig. 4). The molecular interaction between MP polymer and heavy metal ions majorly determines its adsorption behavior (Zhou et al. 2020). Plastic polymer breakdown is hypothesized to trigger metal ions to adhere to the surface of microplastics (MPs) through adsorption. Otherwise, MPs are generally considered unresponsive to metallic ions in water (Fu et al. 2021b). The sorption behavior of Cd, Co, Cr, Cu, Ni, Pb, and Zn elements on plastic resin pellets sampled from southwest England beaches has been investigated (Holmes et al. 2012). According to the findings, ionic sites on the plastic surface provide binding sites for heavy metals, while non-specific interactions are responsible for heavy metal adsorption on neutral zones of MPs. Another study showed that electrostatic interaction between metal cations including Cu^2+^, Ni^2+^, and Zn^2+^ and carboxylate ions on the surface of MPs might be responsible for their adsorption (Tang et al. 2022). The mechanism of heavy metal adsorption on the surface of MPs could be explained in the following three phases (Guo and Wang 2019): (a) heavy metal surface dispersion on the MP surface, (b) the heavy metal pore volume diffusion in MPs, and (c) adsorption on the polar sites.

Research on the adsorption behavior of heavy metals on MPs suggests that the adsorption of Pb^2+^ onto nylon MPs transpires spontaneously and involves an endothermic reaction. The carboxylic groups are generated as a result of nylon degradation, providing binding sites for Pb^2+^ ions and being regulated by the surface complexation process (Tang et al. 2022). Correspondingly, in another research, the mechanism of Pb^2+^ adsorption was mostly based on the interaction of carboxyl and hydroxyl groups, as well as an electrostatic exchange reaction (Fu et al. 2021b). In contrast, one study found that the sorption of Cd^2+^ on MPs may be linked to the sequential exchange process (Guo et al. 2020). The study indicated that distinct chemical moieties on the exterior of MPs, as well as surface sorption and diffusion effects, are related to Cd^2+^ adsorption instead of chemisorption. The impacts of physical weathering of MPs on heavy metal adsorption behavior, such as UV exposure, oxidative reactions, and heat decomposition, have also undergone extensive examination (Rani et al. 2017; Kedzierski et al. 2018; Liu et al. 2019a). Usually, oxygen-containing groups formed during weathering have the propensity to enhance the polarity, porosity, hydrophilicity, and ionic charges on the surface of MPs, which affects the adsorption of heavy metals on MPs (Liu et al. 2019a). The adsorption of heavy metals on PS MPs was examined under different environmental conditions such as UV rays, freshwater, marine water, and air (Mao et al. 2020). The results indicated that the adsorption capacity of metal ions on the surface of PS MPs increases with MP aging, which may increase surface roughness and oxygen atom-containing groups that provide a binding site for them. These findings suggested that weathered MPs pose greater harm than pristine MPs of identical polymer composition because environmental influences generate greater surface roughness and oxygen groups, which give an adequate site for metal sorption. Despite these negative impacts of MP transporting heavy metals, there has been relatively limited investigation on the effects of plastics aging under natural environmental settings. The maturation of MPs in the open environment is influenced by a variety of environmental conditions, including the MP residency period and the concentration of heavy metal ions in that specific site, which causes difficulty when comparing existing data. Hence, it is critical to distinguish between the adsorption behavior of metal ions in natural wreathing and artificial aging in the future.

Existing microorganisms form a biofilm around the MPs, altering the surface shape and physicochemical properties of the MPs and increasing heavy metal adsorption (Rummel et al. 2017). For 28 days, field research was conducted to investigate the impacts of biofilms in an urbanized estuary using suspended plastic pellets (Richard et al. 2019). Upon conclusion of the experiment, it was discovered that elevated levels of metals (Al, Co, Cu, Mn, Pb, and U) on plastic pellets are indicative of a high concentration of metals directly associated with biofilm abundance on MPs. This might be because biofilm formation stimulates the creation of hydrous oxides and active binding sites, as well as the formation of organometallic compounds with metal ions and hydrous oxides, allowing metals to be removed during the separation procedure from biosorption (Rochman et al. 2014; Fu et al. 2021b). However, complicated physical and chemical processes are responsible for the sorption of heavy metals in biofilms. MPs covered with biofilm from a reservoir and a eutrophic urban lake were used for a 1-month experiment to study metal absorption (Guan et al. 2020). The research emphasized that biofilm functional moieties, including phenolic, amino, and carboxylic groups, enhance the sorption of heavy metals onto MPs through processes such as cation exchange, electrostatic interchange, and interaction with functional moieties.

On the other hand, UV light has an essential role in creating biofilm on the surface of MPs, which can increase its lifespan and reduce metal adsorption on the MP surface (Richard et al. 2019). Although the preceding investigations were conducted over a short period, microbial communities change over time. Each microbial community may exhibit some distinctive behavior toward adsorption mechanisms, which influences metal binding on MP surfaces. To further understand the mechanisms involved in metal ion adsorption on MP surfaces, future studies should focus on long-term processes for microbial development on MPs and their effects on heavy metal bioavailability in the aquatic environment.

## Interaction of MPs with aquatic biota

### Microalgae

Microalgae play a pivotal role as the main contributors to the aquatic ecosystem and are also affected by MPs which raises a serious question about aquatic ecosystem existence (Liu et al. 2019a). Because of the size variability of microalgae from micrometers (μm) to millimeters (mm), they have incredible potential to interact with MPs in aqueous conditions.

The mechanism of action (MoA) of MPs in microalgae can shift from the cellular to the communal level, with growth rate suppression being the primary impact (Mao et al. 2018; Liu et al. 2019b; Prata et al. 2020; Wang et al. 2020). The metabolic impacts include the reduction of photosynthesis and the production of reactive oxygen and nitrogen species (ROS and RNS) that cause oxidative stress in algal cells. The MoA interaction between algal cells and MPs can be explained in two ways: (a) interaction through cell walls or/damaged membrane for direct contact that cause cytotoxicity in algal cells (Liu et al. 2019b) and (b) MPs affect the photosynthesis process in microalgae through shading effect (limited availability of light) in the water column (Wang et al. 2020). MPs with particle sizes of < 1 mm can adhere to the surface of microalgae and influence them in a variety of ways: (i) decrease the cell membrane fluidity; (ii) disturb the cell membrane transportation; (iii) influence the availability of nutrients in algal cells, affecting metabolism and growth; (iv) produces negative effects on cell structure, such as cell membrane damage, and plasmolysis (Mao et al. 2018; Liu et al. 2019a). Furthermore, MP adsorption on algal cells can promote the secretion of protein-rich exopolymeric substances (EPS) that facilitate MPs and biogenic aggregation, along with MP surface alterations, which can influence the fate and colonization of microalgae (Shiu et al. 2020). The MPs with particle size < 1 μm can find their way into the algal cell through cell membrane pores (Garrido et al. 2019). To summarize, the MoA of MPs on algae is primarily contingent on size, particularly noticeable with larger MPs causing, photosynthetic stress owing to light obstruction. In contrast, small-sized MPs adsorb through the cell membrane, creating biochemical changes affecting cell growth and functions.

### Molluscs or aquatic floor organisms

Organisms from group mollusca are good bioindicators of ecotoxicity in aquatic environments. Interestingly, bivalve molluscs have suspension-feeding habits because of their proficiency in sieving substantial water quantities and encapsulating small particles together with MPs, efficiently (Rosa et al. 2018). Therefore, MP fibers have been reported in mollusc tissues abundantly, from all over the world.

The uptake of MPs in molluscs can occur either passively or actively. The passive method is a self-occurring process that happens due to their structure and morphology; molluscs exhibit modifications in their feeding organs. The adhesion of sticky particles on the feeding organs is accountable for passive uptake, whereas in the case of active or natural uptake method, selection of particles is triggered by fine alteration in the structure or drifting in feeding organs, such as muscle contractions or movement of cilia or cirri over these organs (Evan Ward and Shumway 2004). In the case of bivalves, uptake of MPs followed two pathways: (A) through gills, MPs can be trapped in the gills as they are the foremost organs, and (B) through inhalation via the siphon, MPs can move towards the oral region and subsequently undergo additional translocation and distribution throughout the entire system. Initially, the uptake of MPs is carried out by the microvilli located on the surface of gills. The adhered particles are transported by endocytosis, responsible for the transport of dust and tiny particles in bivalves. The second pathway includes the activity of the ciliary that guides the MPs into the digestive system and facilitates their journey to the primary and secondary ducts of gastrointestinal tubules. The MPs can reach the circulatory system or hemolymph from both routes, through gills as well as by the digestive gland, and circulate in the entire system (Ding et al. 2018). However, the retention time of MPs in the gut region is highly dependent on the morphological structure of latero-frontal cilia (Møhlenberg and Riisgård 1978). The density of the MPs also plays a crucial role in the retention of MPs; lighter particles can retain for a longer time than heavier ones (Brillant and MacDonald 2000). Moreover, rough or irregular particles of MPs can retain for a longer time than smooth ones (Huvet et al. 2016). Therefore, the wide distribution of MPs in mollusc tissues indicated a red sign for the aquatic food chain because of their close association with predators and human health.

### Fish

Owing to their small size and high resemblance with natural food, MPs are highly consumed by fish species, while the presence of MPs has been documented in diverse aquatic organisms such as zooplankton, bivalves, and copepods (Browne et al. 2008; Cole et al. 2013; Chua et al. 2014; Pal and Maiti 2018a, 2018b, 2019a, 2019b, 2020) (Table S1), but accumulation and distribution of MPs in commercial and highly consuming organisms are expected to cause exposure risk to human population with possible adverse effects over time.

Ingestion of MPs in fish tissues is dependent on the bioavailability and concentration of pollutants in the surrounding water, physiology of the organism, exposure time, and feeding behavior (Akhbarizadeh et al. 2018). The primary route of MPs in fishes is majorly considered through ingestion. The ingested particles are assumed to reside in the intestinal tract, translocated to other parts or fish tissues, and finally eliminated (Fossi et al. 2018). Furthermore, MPs have a propensity to attach to the skin of fish and relocate to different organs, including the liver, gills, and muscles (Su et al. 2019). A study conducted by Wright and Kelly (2017) revealed that ultrafine plastic particles can traverse biological membranes and translocate either via the vascular system or lymphatic pathways, demonstrating the distribution of MPs throughout the fish body.

## Risk characterization and human health risk assessment

**Fig. 5:**
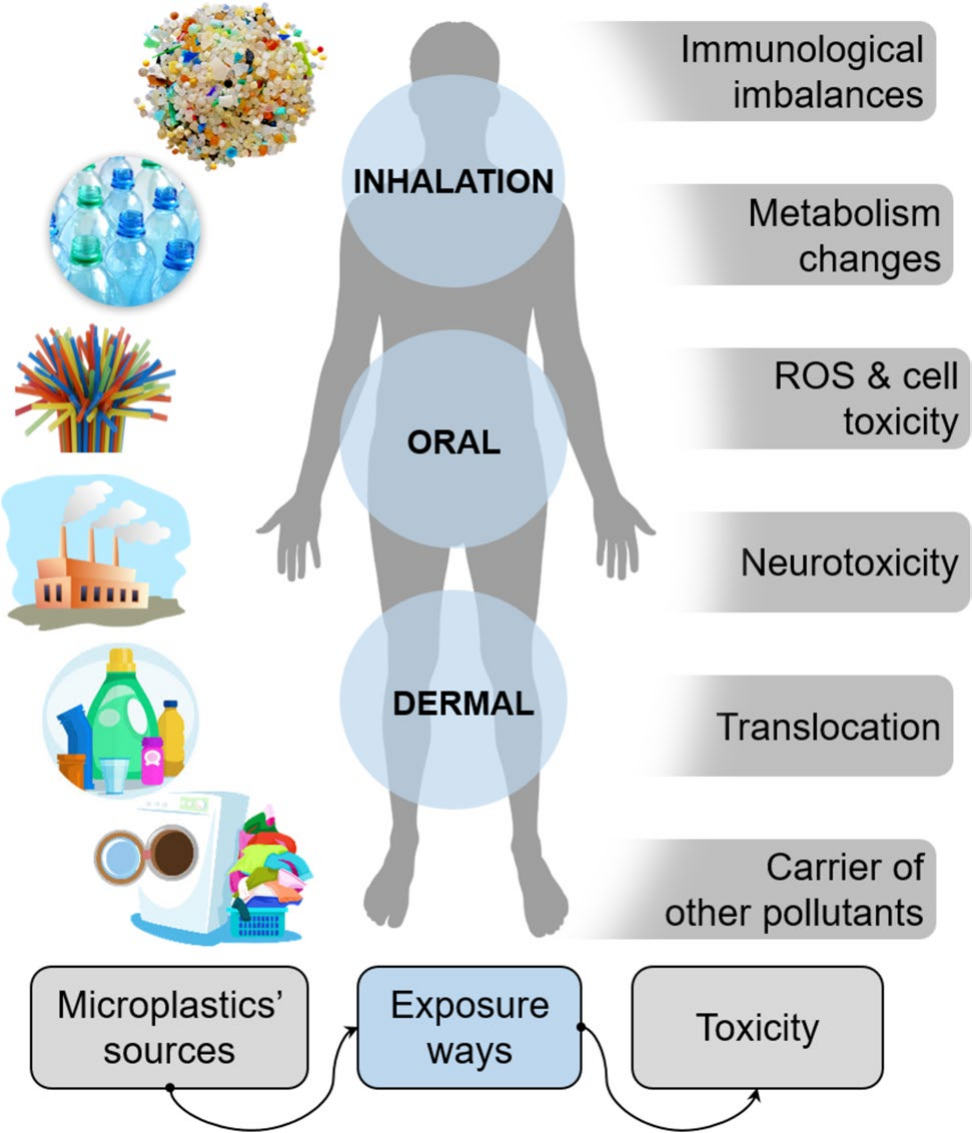
Potential sources, exposure routes, and toxicity routes of MPs/NPs in the human body

The pervasive presence of MPs in the environment raises substantial health concerns, driven by their persistent nature, bioavailability, and potential effects on human physiology. Human exposure to MPs occurs primarily through ingestion, inhalation, and, potentially through skin contact, as illustrated in Fig. 5. These exposures impact various bodily systems, including gastrointestinal, respiratory, reproductive, and neurological, prompting investigations into MP health risks and necessary regulatory responses.

### Routes of exposure and uptake of MPs/NPs

#### Ingestion

Ingestion is a primary exposure route, with MPs entering the gastrointestinal (GI) tract via contaminated food and drink. Common sources include seafood, milk, bottled water, and salt (Mason et al. 2018; Kutralam-Muniasamy et al. 2020; Kwon et al. 2020). Animal studies have shown that MPs accumulate in tissues through ingestion and transfer up the food chain, increasing human exposure risk (Cedervall et al. 2012). MPs have been detected in 81% of global tap water samples, mainly as fibers < 5 mm (Kosuth et al. 2018). In bottled water, particle counts can reach up to 6292 particles per liter (Schymanski et al. 2018; Oßmann et al. 2018). Infants face higher exposure due to frequent use of plastic containers (Zhang et al. 2021a, 2021b), with estimated weekly consumption at 0.1–5 g, roughly equivalent to the weight of a credit card (Senathirajah et al. 2021).

#### Inhalation

Airborne MPs, originating from synthetic fibers, tire wear, and industrial sources, can enter the respiratory tract. The extensive alveolar surface (~150 m^2^) and thin epithelial barrier (~1 μm) allow NPs to penetrate capillaries and enter systemic circulation (Lehner et al. 2019). Autopsies have confirmed MP presence in lung tissues, raising concerns about prolonged respiratory exposure (Amato-Lourenço et al. 2021). Occupational exposure to airborne MPs, such as polystyrene, is linked to increased styrene metabolites in urine, suggesting biomarker potential for inhalation exposure (Persoons et al. 2018).

#### Skin contact

Skin exposure is less studied but occurs through prolonged contact with MPs in cosmetics and textiles. Certain plastic additives, like bisphenol A (BPA), can induce contact dermatitis, though penetration into deeper skin layers is rare (Aalto-Korte et al. 2003). NPs can potentially penetrate the skin via sweat glands, hair follicles, or damaged areas (Yee et al. 2021), but they predominantly accumulate in the outermost skin layers. A recent study used a 3D human skin equivalent model and tested the bioavailability of polybrominated diphenyl ether (PBDEs), a well-known chemical additive present in MPs. The study confirmed that more sweaty skin resulted in higher bioavailability of PBDEs from dermal contact than dry skin (Akpojevwe Abafe et al. 2024).

### Effects of MPs on human organ systems

#### Gastrointestinal tract

The GI tract is vulnerable to MPs, which may disrupt gut microbial communities and trigger inflammation. Higher fecal MP concentrations have been observed in patients with inflammatory bowel disease (IBD) compared to healthy controls (Yan et al. 2022). Animal studies show that MPs contribute to gut dysbiosis and barrier impairment, potentially leading to metabolic disorders, such as non-alcoholic fatty liver disease (Chen et al. 2022). Chronic MP accumulation has also been linked to a heightened risk of colorectal and pancreatic cancers (Oddone 2014).

#### Respiratory tract

The respiratory system is particularly susceptible to MPs and NPs due to its extensive surface area and thin epithelium. Studies in exposed workers have noted links between MP exposure and respiratory conditions like hypersensitivity pneumonitis and obstructive bronchiolitis (Ruder and Bertke 2017). Although causality between MP exposure and lung cancer remains inconclusive, autopsy data confirm MPs in lung tissues, indicating risks associated with chronic inhalation (Amato-Lourenço et al. 2021).

#### Skin and allergic reactions

MPs in consumer products, including cosmetics and textiles, may pose dermatological risks. Cases of contact dermatitis have been attributed to plastic additives, including BPA (Rose et al. 2009). While direct penetration into deeper skin layers is uncommon, MPs can interact with immune cells in the skin, potentially triggering localized inflammation (Heilig et al. 2011).

#### Reproductive system

Research suggests that MPs can cross biological barriers, including the placenta, potentially affecting fetal development. MPs have been detected on both maternal and fetal sides of human placentas (Ragusa et al. 2021). Animal studies indicate that MPs can accumulate in reproductive organs, leading to reduced fertility and gonadal damage, such as ovarian fibrosis and decreased sperm viability (An et al. 2021; González-Acedo et al. 2021).

### Cellular and molecular impacts of MP exposure

This section highlights the combined toxicity of MPs and various environmental pollutants, revealing significant effects on aquatic organisms. Table 3 presents a detailed overview of key studies on synergistic toxicity, including pollutant types, observed biological impacts, mechanisms of toxicity, and potential implications for human health. These interactions often culminate in cellular impacts, where MPs exhibit cytotoxic and genotoxic effects, disrupting cellular functions and posing additional health risks, as discussed below.

**Table 3:** Table on synergistic toxicity of MPs and co-pollutants in aquatic organisms

MP type	Co-pollutant	Impacted species	Observed synergistic effect	Mechanism of toxicity	Human health implications	References
PE	Cadmium (Cd)	Zebrafish, protozoan	Bioaccumulation, metabolic disruption, microbiota imbalance	Facilitates Cd uptake, causing DNA damage, inflammation, apoptosis	Cd-laden MPs could transfer via the food chain, posing metabolic risks	Zhang et al. (2024)
PVC	Cd	Mussel	Cd bioaccumulation in digestive gland, increased oxidative stress markers	Induces oxidative stress via upregulation of antioxidant markers	Potential risk of oxidative stress for seafood consumers	Li et al. (2020)
Unspecified MPs	Mercury (Hg)	European seabass	Enhanced neurotoxicity, oxidative stress in brain and muscle tissues	MP-mediated Hg bioaccumulation disrupts enzymes and neural pathways	Hg bioaccumulation poses risks to top predators and food safety	Barboza et al. (2018)
Unspecified MPs	Copper (Cu)	Zebrafish	Impaired swimming, neurological effects, reduced survival rate	Behavioral disruption through AChE inhibition	Risks to fish populations and predator-prey dynamics; neurotoxicity concerns	Santos et al. (2021)
PA	Bisphenol A (BPA)	Zooplankton	Reduced BPA bioavailability and toxicity	Sorption of BPA reduces aqueous phase concentrations	Lower BPA uptake mitigates toxicity; minimal bioaccumulation impact	Rehse et al. (2018)
PS	Triphenyltin (TPT)	Marine diatom	Reduced TPT toxicity due to increased sorption by PS	Smaller particles enhance pollutant adsorption, reducing bioavailability	Potential mitigation of pollutant toxicity in marine food webs	Yi et al. (2019)
Unspecified MPs	Tributyltin (TBT)	*Rotifer*	Reproductive disruption, oxidative stress	Increased ROS and disrupted antioxidant activity; multi-generational effects	Risks of oxidative stress and reproductive impacts	Yoon et al. (2021)
PE	Crude oil (polycyclic aromatic hydrocarbons, PAHs)	Arctic copepod	Reduced feeding, increased PAH bioaccumulation with dispersant use	Behavioral stress; chemical dispersant promotes PAH uptake	Trophic transfer of PAHs affects food quality for higher organisms	Almeda et al. (2021)
PS microbeads	3-Nitrobenzanthrone (3-NBA) and benzo[a]pyrene (BaP)	Rainbow trout cells	Increased DNA damage in intestinal and gill cells	Genotoxicity via DNA adduct formation and oxidative stress	Potential for bioaccumulation and genotoxic risk in food webs	Bussolaro et al. (2019)
PE and PS microspheres	Polychlorinated biphenyls (PCBs)	Norway lobster	Limited PCB uptake in tissue; minimal nutritional impact	Chemical partitioning limits PCB desorption from MPs to tissues	Lower PCB transfer suggests minimal risk, but real-world implications for humans need further study	Devriese et al. (2017)
HDPE	Chlortoluron (herbicide)	Pacific oyster	Decreased shell growth, altered valve activity	Disrupts physiological activity, impacts development	Potential for reduced oyster growth impacting aquaculture and food supply; potential bioaccumulation effects	Bringer et al. (2021)
Unspecified MPs	Butylated hydroxyanisole (BHA)	Zebrafish larvae	Developmental toxicity, reduced hatching, increased malformations	Disturbs thyroid hormones and lipid metabolism	Risks of developmental and metabolic disorders	Zhao et al. (2020)
PSNPs	Pharmaceuticals	Marine fish cell	Enhanced toxicity of pharmaceuticals	Increased antioxidant activity and oxidative stress	Cumulative toxic impacts on marine organisms and possibly humans	Almeida et al. (2019)

*HDPE* high-density polyethylene, *PSNPs* polystyrene nanoplastics

#### Cytotoxicity and genotoxicity

In vitro studies indicate that MPs, particularly polystyrene nanoparticles (PS-NPs), induce cytotoxicity and genotoxicity depending on particle size and surface modifications (Brachner et al. 2020). MP exposure leads to DNA damage, sister chromatid exchange, and increased micronuclei formation in cells, especially in occupational settings with high styrene exposure (Teixeira et al. 2010). MP bioactivity is further modulated by the protein corona that forms upon contact with biological fluids, varying across physiological states (Ahsan et al. 2018).

#### Oxidative stress and inflammation

MPs are known to induce oxidative stress, where excessive reactive oxygen species (ROS) production surpasses cellular antioxidant defenses, leading to tissue damage (Werder et al. 2018). This oxidative stress, combined with inflammation, can exacerbate conditions such as asthma and other inflammatory diseases, especially in settings with prolonged or high-level exposure. Recent researches on cellular exposure to MPs /NPs reveal that these particles can induce various responses, including cytotoxicity, genotoxicity, inflammation, apoptosis, and oxidative stress (Brachner et al. 2020). In vitro studies have primarily focused on PS as a model polymer, despite PS not being among the most prevalent plastics globally. This indicates a gap in data for other widely found polymers, especially PET and PVC, which are also significant contributors to human exposure and potential toxicity (Park et al. 2020).

Particle size and surface modifications are critical factors influencing cytotoxicity and inflammatory responses. Research has examined particles ranging from nano- to millimeters, often observing stronger biological interactions with smaller particles like NPs. These particles can more easily penetrate cellular membranes, leading to the generation of ROS, which exacerbates oxidative stress and inflammation (Ahsan et al. 2018). However, studying these effects presents challenges due to difficulties in isolating and extracting MPs from environmental or biological samples, complicating accurate exposure and dosage assessments. Furthermore, data on long-term exposure effects remain limited, despite the likelihood that plastic particles could bioaccumulate and persist in human tissues over a lifetime, raising concerns about chronic, low-level exposure impacts (Brachner et al. 2020).

Upon entering the body, NPs often interact with biomolecules such as proteins, lipids, and sugars, potentially altering their properties. For instance, when NPs contact biological fluids like serum or plasma, they form a “protein corona”—a dynamic layer of adsorbed proteins that modifies their surface characteristics (Park et al. 2020). This protein corona varies with physiological factors, such as stress, diet, and health status, potentially influencing how cells recognize these particles, which could alter toxicity. However, the full extent of these effects remains underexplored.

Currently, in vitro studies have not sufficiently explored personalized cellular responses based on specific protein corona compositions, leaving questions about how these variations affect particle behavior and toxicity. More extensive research on diverse polymers and tailored studies on individual physiological conditions is essential to better understand the long-term cellular impacts of MPs and NPs on human health.

### Influence of chemical properties of MPs on human health

The interaction between MP chemical composition and human health adds complexity to risk assessment. MPs contain both inherent chemicals (e.g., polymers and additives) and adsorbed environmental pollutants, which can be harmful when ingested or inhaled.

#### Effects of encapsulated chemicals in MPs

Additives in plastic production, such as halogens, pigments, and flame retardants, can be toxic. These chemicals have been linked to cellular toxicity, genotoxicity, and endocrine disruption (Winiarska et al. 2024). For instance, BPA, commonly used in plastic packaging, can mimic natural hormones and disrupt the endocrine system (Olea-Serrano et al. 2002) (Table 3). The cellular toxicity of MPs, particularly of smaller particles, increases with decreasing size and higher concentrations, making particle characteristics crucial in toxicity assessment (De Souza Machado et al. 2018; Winiarska et al. 2024).

#### Effects of chemicals adsorbed on MP surfaces

MPs can adsorb pollutants from their surroundings, concentrating these contaminants on their surfaces by up to 10^6^ times the surrounding environment levels (Teuten et al. 2007). This adsorption capacity depends on factors like MP size, shape, and surface chemistry, as well as environmental conditions (Fernández et al. 2020). MPs facilitate pollutant transport across environments, with PAHs demonstrating strong sorption to MPs in high-salinity waters, thereby enhancing their bioavailability in marine food chains (Sun et al. 2009). Experimental studies indicate that MPs and NPs can disrupt red blood cells, with smaller particles causing notable shape changes and reduced cell flexibility (Barbul et al. 2018).

**Fig. 6:**
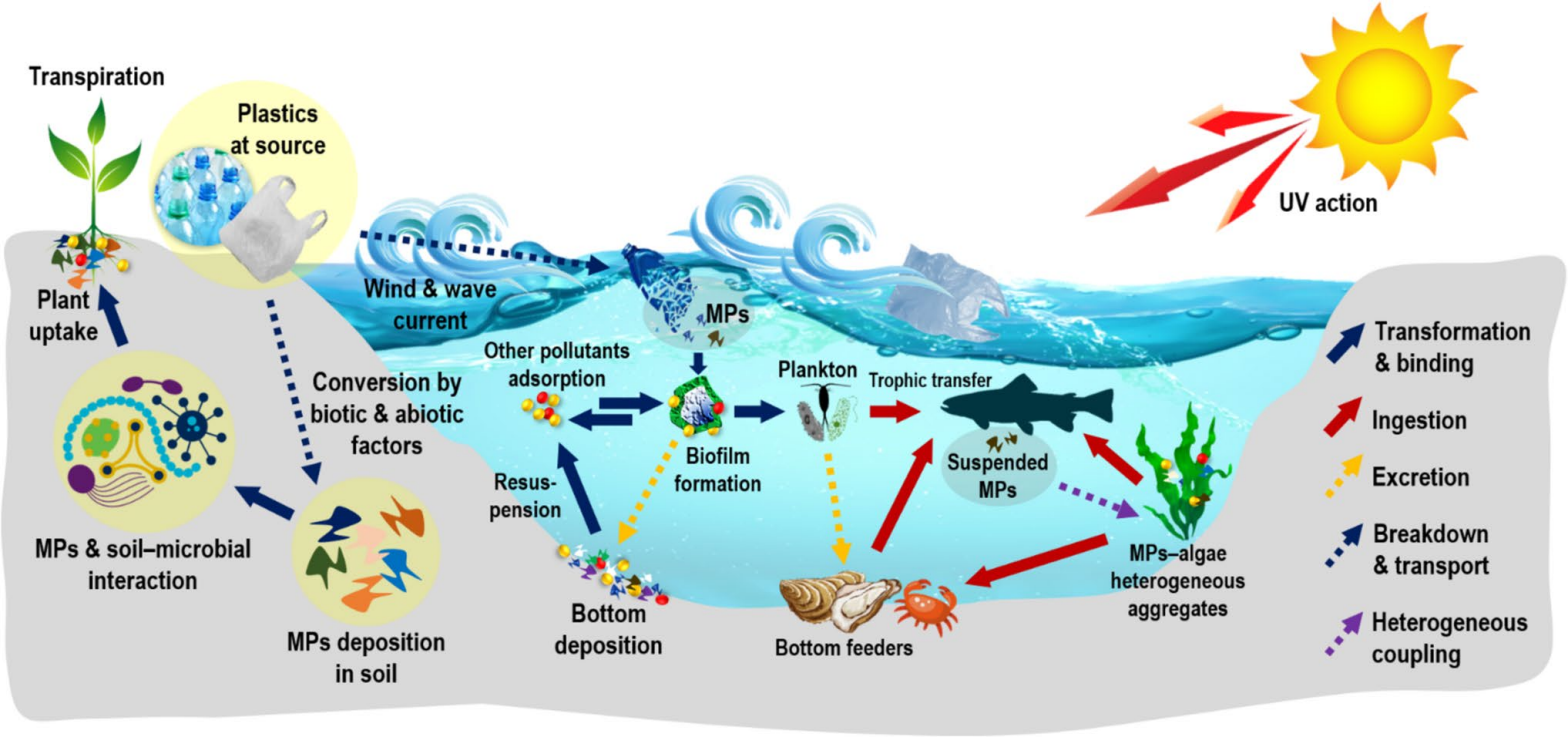
A schematic description of bioaccumulation and effects between MPs/NPs and other organic and inorganic pollutants in the terrestrial and aquatic ecosystem

In terrestrial ecosystems, MPs and NPs may translocate into plant tissues, entering the food chain. MP ability to transport pollutants, coupled with their persistence in plants like rice, raises concerns about MP mobility in agriculture and potential health impacts from crop contamination (Liu et al. 2022) (Fig. 6).

The exposure pathways, biodistribution, and cellular effects of MPs indicate substantial implications for human health. Ingestion and inhalation represent the primary routes, with MPs detected in multiple tissues, including the gastrointestinal and respiratory tracts, and even placental tissue. Evidence points to potential risks in reproductive, respiratory, and gastrointestinal health, with MPs linked to oxidative stress, inflammation, and possible carcinogenic effects. Reproductive toxicity is particularly concerning, with studies showing placental transfer and gonadal accumulation, necessitating further research into chronic health impacts. Occupational studies underscore respiratory risks, associating styrene and plastic-derived compounds with respiratory disorders and carcinogenesis. Emerging cellular data, including DNA damage and oxidative stress, highlight the urgency for more rigorous studies on long-term, low-dose exposure. Protein corona formation on MPs complicates their cellular interactions, potentially enhancing or modifying their bioactivity and toxicity.

## Sampling, identification, and quantification of MPs in water and sediment matrix

### Methods for MP sampling from water

#### Neuston, Manta nets, and plankton nets

These nets are widely used, facilitating large-volume water sampling and yielding abundant MPs for further testing. Advantages include ease of use, compatibility for comparative studies across different locations, and capability for high sample yields (Prata et al. 2019). However, limitations involve high equipment costs, the need for a boat, potential contamination from vessel or tow ropes, time-intensive processes, and a detection threshold of 333 μm. Another popular method includes the usage of plankton nets, which are advantageous for detecting smaller particles, with a detection limit of 100 μm, and they enable quick, medium-volume sampling (Prata et al. 2019; Egessa et al. 2020). Despite their ease of use, the equipment is expensive, boat access is required, and static sampling relies on water flow, which may lead to clogging or equipment breakage. Additionally, they sample lower water volumes compared to Manta trawls.

#### Sieving and pumping

This method requires minimal equipment and does not require a boat, making it accessible for collecting moderate water volumes. However, it is labor-intensive and time-consuming, often involving manual water transfer via buckets. In this approach, approximately 5 L water samples were collected from the Wei River and repeatedly filtered through a fine 75-μm stainless steel sieve to enhance the capture of smaller MPs (Ding et al. 2019). In another study, 5 L surface water samples were collected from the Goulburn River, and collected water was filtered through a 20-μm nylon membrane sieve, enhancing the detection of smaller MPs (Nan et al. 2020). Compared to the previous field-based 75-μm stainless steel sieve method, this method offers greater precision and contamination control but requires laboratory facilities and specialized equipment.

Pumps facilitate large-volume water sampling with adjustable mesh sizes, offering efficient sample collection despite needing energy, equipment, and facing contamination and portability challenges. Using this approach, about 30 L water samples were pumped through a 330-μm nylon mesh for MP collection from the transboundary Ganga river (Napper et al. 2021). In another study, a pumping method with 300 μm and 100 μm sieves was used, and it was found that for monitoring environmentally relevant size fractions of microlitter, a mesh size of 100 μm or smaller is recommended for the Baltic Sea (Setälä et al. 2016).

#### Alternative surface water sampling methods

An alterative water sampling method includes MuMi sampler; 3-D printed hydrodynamic device designed for high-resolution MP sampling, equipped with interchangeable filters; and a flow meter for real-time volume monitoring. Unlike traditional Manta nets, MuMi offers enhanced precision and ease of handling due to its compact design and stabilizing fins, minimizing turbulence. Although it requires a 12 V power source, its real-time data capability makes it ideal for detailed MP analysis (Montoto-Martínez et al. 2022). In another study, two newly developed MP sampling systems (New Types I and II) were used for the water sampling, which provides real-time, in situ size fractionation through sequential Y-shaped filters. The New Type I operates in a mobile configuration, sampling as the vessel travels, while New Type II functions in a stationary mode (Du et al. 2022). Compared to the MuMi sampler, which allows filtration to 50 μm without sequential fractionation, these systems enable more comprehensive size distribution data across larger spatial areas. However, the new systems require extensive equipment and high power, presenting logistical challenges relative to the MuMi sampler portability and suitability for fine-scale particle analysis.

A study conducted in the Kattegat/Skagerrak area, Denmark, used UFO (Universal Filtering Objects) system for MP collection from surface water. This method allows to collect water samples at 3 m depth, filtering water sequentially through 300-μm and 10-μm stainless steel filters without additional pumping (Gunaalan et al. 2023). Unlike MuMi and New Type I/II systems, which target surface waters, the UFO system captures particles from a deeper, turbulent zone, offering high filtration precision and reduced operational complexity. This system is advantageous for studies targeting subsurface MPs with minimal setup, while MuMi and New Type I/II provide broader surface coverage and finer fractionation capabilities.

### Sampling of MPs in sediments

Sediment eventually serves as a sink for the pollutants. In the sediment matrices, aquatic biota interacts extensively with MPs; hence, its quantification is of utmost importance. Sampling, extraction, and quantification are the three main aspects of the analytical process that need to be considered for accurately measuring MPs in sediment samples (Hanvey et al. 2017). Although there is rapid development in MP research, an inconsistency in the sampling and extraction techniques for MP estimation in sediments can be seen. The lack of universally validated methods has given rise to a variety of analytical approaches, impeding a comprehensive interpretation of current results on a broad scale. The research challenge lies in the complexity of selecting an appropriate method for MP sampling in sediment due to the wide range of available methods. In this section, we aim to consolidate the most pertinent methods for sampling MPs from sediment. Furthermore, the uneven distribution of MPs in sediments necessitates the need to follow a proper sampling method.

Only an appropriate sampling method will help to produce a reliable result since the concentration of MPs differs depending on the sampling area and the depth covered. Therefore, the choice of sampling technique should consider the matrices designated for sampling and the size constraints of the intended MPs (Wang and Wang 2018). Usually, the three sampling techniques, viz., selective sampling, bulk sampling, and volume-reduced sampling techniques, are employed for the collection of MP sample from the aquatic environment. Among these, bulk sampling is generally employed for collecting sediment samples (Tsang et al. 2017). Samples are mainly collected from the beach, seabed, or superficial depths of the seashore. Collection of samples from beaches is done at a depth of 2–5 cm using stainless spoons, spatula, or hand shovels. Sampling from superficial sediments and seabed requires bottom trawls or specialized equipment, such as a grab sampler and box corer (Prata et al. 2019).

Another study developed a Sediment-Microplastic Isolation (SMI) unit, which is a compact, contamination-controlled approach for MP extraction from sediments, integrating sequential filtration and density separation within a single device (Coppock et al. 2017). Unlike traditional bulk and selective sampling methods that often require multiple devices and extensive labor, the SMI unit provides efficient particle isolation with minimal cross-contamination risk. Although optimal for laboratory settings with a controlled environment, its limited sediment capacity and dependence on external stirring restrict field applicability. This makes the SMI unit highly suitable for precise lab-based MP analysis, whereas traditional methods remain preferable for larger-scale field sampling.

### Separation strategy of MPs from samples

After sampling, MPs need to be separated before quantification and characterization. The most commonly used method for separating MPs is sieving. Sieves of different mesh sizes are used to isolate the MPs from the matrix; however, the method is not sufficient for small-sized MP particles. Different laboratory-based extraction techniques are further required for MP effective separation (Hanvey et al. 2017). Density-based separation in combination with filtration is used to isolate small MP particles (< 1 mm) from samples. Different high-density solutions, such as NaCl, NaI, and ZnCl_2_, have been used to make the separation process efficient. Sometimes, the organic matter present in the sample interferes with the accurate estimation of MPs. These organic contents are separated from polymers using a matrix removal process where the samples are digested using different acids, alkalis, and oxidizing agents (Prata et al. 2019). Different active and passive separation methods are typically used for smaller-sized MP particles (< 1 μm). Active separation of MPs is achieved using the field flow fractionation (FFF) method, where MP particles are subjected to different magnitudes of externally applied field forces for successful separation. The passive separation utilizes the hydrodynamic chromatography (HDC) method, which uses hydrodynamic and surface forces for particle separation (Fu et al. 2020).

### MP identification and quantification

#### Visual inspection method

The visual inspection technique involves the identification and classification of MPs based on their morphological traits, such as color and dimensions, through direct observation or using a microscope (Table S2). Sorting MPs with the naked eye and optical microscope is the predominant method used for their quantification. The approach is applicable for large-scale sample volumes, particularly in instances where costly analytical instruments are not readily available. However, the method is subjective and may produce large variations between examiners. The probability of errors increases as MPs with lesser diameter are easily miscounted or overlooked. In addition, the morphological changes in the weathered MPs make visual identification even more difficult (Crawford and Quinn 2017). To overcome the errors in MP detection in sediments, researchers propose using fluorescence microscopy with Nile Red (NR) staining for MPs (Kukkola et al. 2022; Meyers et al. 2022; Bakir et al. 2023). Alternative techniques including scanning electron microscopy and laser confocal microscopy can assist in mitigating the limitations of visual inspection methods and precisely identify MPs. However, it is still hard to identify MPs of size less than 100 μm using optical microscopes (Song et al. 2015) and need more effective and reliable technologies for MP detection.

#### Thermal analytical method

##### Pyrolysis gas chromatography-mass spectrometry

Pyrolysis gas chromatography-mass spectrometry (Pyr-GC–MS) is a detrimental analytical method that identifies the type of MPs based on the analysis of their thermally degraded volatile organic products (Fries et al. 2013). Individual MP particles are introduced into a reaction tube where the pyrolysis of the same takes place (Table S2). The vaporous byproducts emitted during the pyrolytic process are subsequently cold-injected and conveyed to the GC column. The characteristic spectra of the pyro-products obtained for unknown samples are compared with the available spectra database made for known polymers that enable the identification of MPs (Käppler et al. 2018). The method requires minimal sample pre-treatment and provides detailed information about the chemical composition and other organic additives present in the MPs. Besides, it remains impervious to variations in size, morphology, and additional organic or inorganic impurities linked with the specimen (Dümichen et al. 2017). However, the technique is ill-suited for examining extensive sample volumes, as it allows the analysis of only one particle per cycle, which requires around 30 to 100 min. Also, another major limitation of the technique is its inability to provide information related to the particle size of the MPs (Käppler et al. 2018).

##### Thermal extraction desorption gas chromatography-mass spectrometry (TED-GC-MS)

Thermal extraction desorption gas chromatography-mass spectrometry (TED-GC-MS) involves thermo-gravimetric analytical (TGA) solid-phase extraction and thermal desorption gas chromatography-mass spectrometry (TDS-GC–MS). The specimen is initially subjected to heating on a TGA balance at temperatures reaching up to 1000 °C. Following this, the decomposed products undergo adsorption on solid-phase reagents and are then conveyed to a thermal desorption unit (TDU). In TDU, the desorption of deteriorated products is attained through temperature escalation, subsequently separated via a chromatographic column, and subjected to analysis employing mass spectrometry (Dümichen et al. 2017). The method saves analysis time for large sample sizes and is suitable for samples that have high mass (up to 100 mg) (Elert et al. 2017).

Among the mentioned analytical methods, Py-GC-MS and TED-GC-MS, TED-GC-MS is non-destructive and faster and allows for the analysis of multiple samples in a single run. However, for complex samples such as sediment, Py-GC-MS may be more suitable because it provides more detailed and specific information about the polymer types without overlap between materials. Despite being slower, Py-GC-MS is ideal for identifying the polymer types of MPs in diverse sediment samples.

##### Differential scanning calorimetry

Differential scanning calorimetry (DSC) is a thermal-based analytical method that identifies the polymers based on the chemicals released during the melting of the MP samples. The method is primarily utilized to investigate thermal characteristics of the sample (Rodríguez Chialanza et al. 2018). It is one of the simplest and cheapest analytical methods; however, the sample needs pre-treatment before its analysis as the DSC signals vary with the size of the MP particles. The larger particles pose challenges to the analysis due to their higher mass-to-surface area ratio in contrast to the smaller particles, leading to disruptions in the analytical process (Huppertsberg and Knepper 2018). In addition, any additives or impurities present in the MPs can influence the transition temperature, thereby resulting in inaccurate analysis results.

#### Vibration spectral method

##### Fourier transform infrared spectroscopy 

Fourier transform infrared (FTIR) spectroscopy has emerged as the most commonly used method for the chemical characterization of MPs (Table S2). The unique infrared spectrum generated by the FTIR for a particular chemical bond helps in identifying the unknown material. The different bond compositions of an unknown material generate specific spectra, which are then compared with the known reference spectra for polymer identification (Li et al. 2018, 2024). Besides, the FTIR additionally furnishes comprehensive insights into the physicochemical deterioration of MPs by scrutinizing their oxidative magnitude. Different optimized methods of FTIR, viz., attenuated total reflectance- FTIR (ATR-FTIR), micro-FTIR, and focal plane array- FTIR (FPA-FTIR), have been used for precise and accurate estimation of MPs. ATR-FTIR is generally used in the identification of large-sized (> 500 μm) and irregularly shaped MP particles. However, the processing and separation of MPs from the filters may result in the loss of the smallest MP particles (Shim et al. 2017). Unlike ATR-FTIR, micro-FTIR allows the analysis of smaller MP particles (> 10 μm) directly on the filter paper. As a result, the chances of external contamination and the time required for particle sorting are reduced (Chen et al. 2020b). The incorporation of FPA detectors in FTIR even offers the analysis of the whole surface area of the filter containing MP particles (< 20 μm) with a high spatial resolution (5.5 μm in reflectance and 1 μm in ATR mode). The method is an excellent choice for identifying MPs as it is neither sensitive to the thickness of MPs nor is interfered with by contaminants or filter membranes. Compared with other FTIR techniques, the method is much quicker in detecting MPs; however, the technique is more power- and cost-intensive (Huppertsberg and Knepper 2018).

##### Raman spectroscopy

Raman spectroscopy is another spectral method most widely used in identifying the chemical species of the sample by mapping the frequency shifts of the scattered lights from the sample. Monochromatic laser beams are irradiated onto the MP particles that generate a different frequency of backscattered light as a result of absorption, reflection, or scattering depending on the atomic and molecular structure of the sample (Crawford and Quinn 2017). A unique spectrum is produced for each polymer as a consequence of this Raman shift. Micro-Raman enables the identification of MPs as small as 1 μm and, in some cases, even down to 500 nm in high spatial resolution, unlike other conventional spectroscopic techniques (Ribeiro-Claro et al. 2017). Besides, it even allows the analysis of moist samples. However, the accuracy of the method is easily influenced due to the existence of additives, colorants, or other contaminants attached to MPs. Also, the low signal-to-noise ratio creates problems in spectrum analysis (Araujo et al. 2018).

Among the aforementioned vibration spectral methods, the FTIR method has been predominantly used for MP detection in water samples because it detects vibrational modes of polymer functional groups through infrared absorption, which is minimally affected by water. However, the usage of Raman spectroscopy for MP identification in complex matrices such as sediment is mostly preferred because it relies on the inelastic scattering of photons (Raman effect) to identify polymeric structures. Its ability to resolve smaller MPs (down to 1 μm) and its resistance to interference from pigments or mineral particles in sediment make it highly suitable for such analyses.

#### Other analytical methods

##### Scanning electron microscope (SEM)

A scanning electron microscope (SEM) uses a high-intensity electron beam to scan the sample (Table S2). The images were produced to provide information about the sample morphology and other surface details at very high magnifications. These high-resolution images are utilized to examine MP surface morphology which helps differentiate them from other organic and inorganic impurities (Wang and Wang 2018). In addition, the fractures, grooves, cracks, and pits developed on MPs under weathering can also be easily analyzed using SEM. Besides, the interaction between the sample and electron results in the generation of characteristic X-ray photons, which are specific to certain elements. An energy dispersive spectroscopy (EDS) detector can easily differentiate these X-rays. Hence, in-depth insights into the elemental composition and additional inorganic enhancements within the MPs can be readily acquired through the simultaneous application of SEM and EDS. This process further aids in the differentiation of plastic and non-plastic components (Crawford and Quinn 2017). Nevertheless, the method is not suitable for handling a large number of samples due to the considerable time and effort required in sample preparation.

##### Hyperspectral imaging

Hyperspectral imaging (HSI) is a non-destructive analytical method widely used for detailed sample analysis (Faltynkova and Wagner 2023; Goyetche et al. 2023; Ali et al. 2024). This technique generates spectral information through interactions between incident light of varying wavelengths and the chemical species in a sample (Ye et al. 2022), producing spectral signatures that vary depending on the samples’ physical and chemical composition. HSI enables direct visualization of samples, aiding in the identification of polymer types in MPs (MPs) by analyzing their shape, size, and composition (Karlsson et al., 2016). It has been applied to MPs in various environmental samples, including soil (Ai et al. 2022), marine sediments (Goyetche et al. 2023), seawater (Shan et al. 2019), freshwater (Fiore et al. 2022), urban wastewater treatment plants (Nguyen et al. 2021), and fish tissues (Zhang et al. 2019). HSI shows significant potential in MP research, especially when combined with classifiers like support vector machine (SVM) and partial least squares discriminant analysis (PLS-DA), which enhance detection accuracy across a broad MP size range down to 0.1 mm. Further, dimensionality reduction techniques, such as principal component analysis (PCA), improve data handling, enabling efficient processing of high-dimensional spectral data. The successful application of these methods in different wave ranges, from near-infrared (NIR) to short-wave infrared (SWIR), enhances MP detection accuracy (Serranti et al. 2018; Shan et al. 2018), which is especially useful for mapping polymer types in complex samples and understanding MP distribution in various environments.

Despite its potential, HSI faces challenges in identifying smaller MPs, particularly particles below 0.1 mm, due to weak signal strength and increased noise from surrounding materials. Reflectance imaging, a common technique in HSI, is particularly affected, although preprocessing techniques such as standard normal variate (SNV) and mean centering (MC) have shown some promise in distinguishing MPs within complex mixtures (Shan et al. 2018). The non-destructive nature of HSI is advantageous for analyzing MPs in mixed environmental samples, as it preserves sample integrity, enabling a more accurate assessment of MPs in natural settings. Targeted wave ranges, such as 900–1700 nm and 1000–2500 nm, help isolate the spectral signatures of MPs more effectively. However, the complex operation and data processing requirements limit its widespread application, compounded by issues such as low-quality images from electron microscopes and low scanning frame rates (Shan et al. 2019). Ultimately, HSI captures spectral signatures based on the unique chemical bonds in MP polymers. In sediment and water samples, MPs reflect light differently due to their composition, such as C-H bonds in polyethylene or polypropylene, allowing differentiation from organic materials. By analyzing these spectral fingerprints, HSI can distinguish MPs from natural particles, even at small sizes, enhancing identification in complex environments. Nonetheless, challenges persist in achieving high resolution and accurate data extraction, as interference from surrounding matrices can limit effectiveness, underscoring the need for further refinement in classifiers and preprocessing techniques to fully harness HSIs capabilities.

### Selection of analytical methods based on polymer type

The choice of analytical method is critical for accurate MP identification, particularly when distinguishing between polymer types. FTIR and Raman spectroscopy are preferred for characterizing polymer-specific functional groups, with FTIR well-suited for water samples due to minimal interference and Raman spectroscopy ideal for sediments, as it can resolve smaller MPs (down to 1 μm) and is less affected by pigments or minerals. Thermal methods like Pyr-GC–MS provide detailed polymer composition data without interference from particle shape or size, making them particularly useful for complex matrices, though they require more time per sample. For samples where morphology and surface structure provide essential information, SEM-EDS and HSI offer insights into both elemental composition and surface degradation patterns. Utilizing a combination of these methods allows for robust polymer-type identification across diverse environmental matrices, ensuring more comprehensive MP characterization.

## Conclusion and future recommendations

This review systematically explored the pathways of MP formation in aquatic systems, their interactions with environmental contaminants, impacts on biota, and implications for human health risk. Our analysis underscores the pervasive and growing presence of MPs in aquatic environments, driven by factors such as UV radiation, temperature fluctuations, and chemical interactions that facilitate polymer fragmentation and chain scission. These degradation mechanisms are further influenced by environmental variables like low pH and high salinity, accelerating the transformation of larger plastics into MPs.

Advances in sampling and analytical techniques, such as coupling Nile Red staining with fluorescence microscopy, have enhanced the detection of MPs in sediments, although comprehensive studies examining the full range of polymer properties are still required for accurate MP identification and quantification. The adsorption of contaminants on MPs is largely regulated by polymer type and physicochemical conditions (e.g., pH, salinity), with weathering-induced morphological changes increasing the availability of sorption sites. Contaminants bind to MPs through various mechanisms, including hydrophobic and electrostatic interactions, hydrogen bonding, and Van der Waals forces, though laboratory-based findings often lack alignment with the complexities of natural ecosystems.

This review also explores the modes of action of MPs within aquatic food webs, from their interference with photosynthetic processes in microalgae to their bioaccumulation in molluscs and translocation within fish. The buoyancy and density of MPs significantly influence their interaction with pelagic versus benthic organisms, highlighting the importance of tailoring environmental assessments to MP characteristics. The detection of MPs in human stool underscores an unintentional route of human exposure, potentially contributing to a spectrum of health issues, from inflammatory and immune disorders to respiratory and gastrointestinal concerns. Current findings underscore that MPs, particularly in high concentrations or in vulnerable populations, may contribute to adverse health outcomes, yet further studies are needed to clarify these associations.

In alignment with our objectives, this review identifies critical factors influencing MP formation, examines their pathways and modes of action across trophic levels, evaluates their potential risks to human health, and assesses methodological advancements in their identification and quantification in aquatic environments. A more nuanced understanding of MP ecological and health implications will be essential in developing comprehensive risk assessments and management strategies.

### Future recommendations

Despite extensive research in this domain, significant gaps persist in the available data. Addressing these knowledge deficiencies requires further exploration of the following issues:

1. First, advancing cost-effective, user-friendly methods for MP sampling and identification, especially in sediment, water, and biological matrices, is critical for consistent and accessible research. Techniques like fluorescence labelling show promise but remain complex and costly.
2. In the actual environment, MPs break down in a more complex way than in controlled lab conditions. Thus, for laboratory investigations, integrating diverse degradation techniques is crucial for achieving both consistency and controllability while simulating the environmental breakdown of MPs. Furthermore, it is crucial to understand how MPs interact with other pollutants at different degradation stages, in varying environmental conditions.
3. Additional research is essential to comprehensively understand the ecotoxicological impacts of MPs under realistic environmental conditions and concentrations. Current toxicity tests employ artificial plastic particles with predetermined size fractions and smooth surfaces on target organisms, which deviates significantly from real-world scenarios. The small size of MPs (or NPs) makes it challenging for researchers to accurately measure their concentration and evaluate their toxicity in organisms, thus turning risk assessment into a tough challenge.
4. Details regarding the trophic transfer of MPs and linked pollutants in multitrophic aquatic food webs remain constrained. The current focus of trophic transfer studies for MPs primarily involves laboratory-feeding-based secondary food chains, which may not accurately reflect the complexity of real biological systems. In reality, higher trophic level predators possess a stronger capacity to bypass the impact of contaminants compared to lower prey. Consequently, there is a crucial necessity to establish comprehensive experimental conditions for scrutinizing the biological impacts of MPs, especially on apex predators and, consequently, humans.
5. Most of the studies are focused on the physical characteristics of plastic objects in certain tissues of aquatic biota and the consequent negative environmental impacts. MPs are not likely to induce gastrointestinal blockage in humans; nonetheless, understanding the process of their movement to non-digestive organs may give insight into human health risk assessment.

## Supplementary information

ESM 1 (DOCX 398 kb)

## Data Availability

Not applicable
