# Supplementary material for: Microplastics in aquatic systems: A comprehensive review of its distribution, environmental interactions, and health risks
Source: Environ Sci Pollut Res Int. 2024 Dec 13;32(1):56–88. doi: 10.1007/s11356-024-35741-1 (PMC11717821; doi:10.1007/s11356-024-35741-1)
Supplement: Supplementary file 1 — (DOCX 398 kb) [file 11356_2024_35741_MOESM1_ESM.docx]

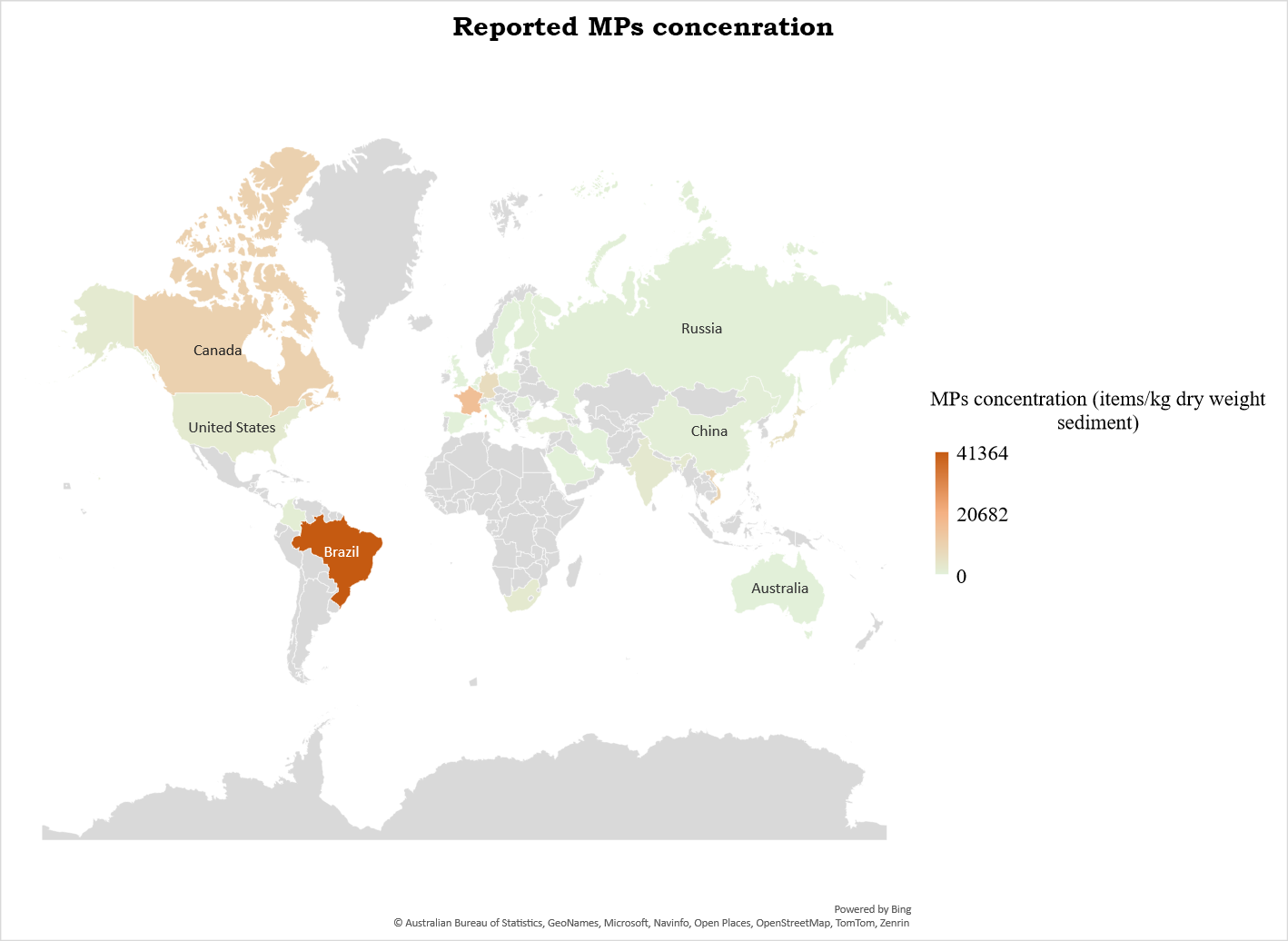


**Fig. S1: Worldwide major countries affected with MPs pollution**


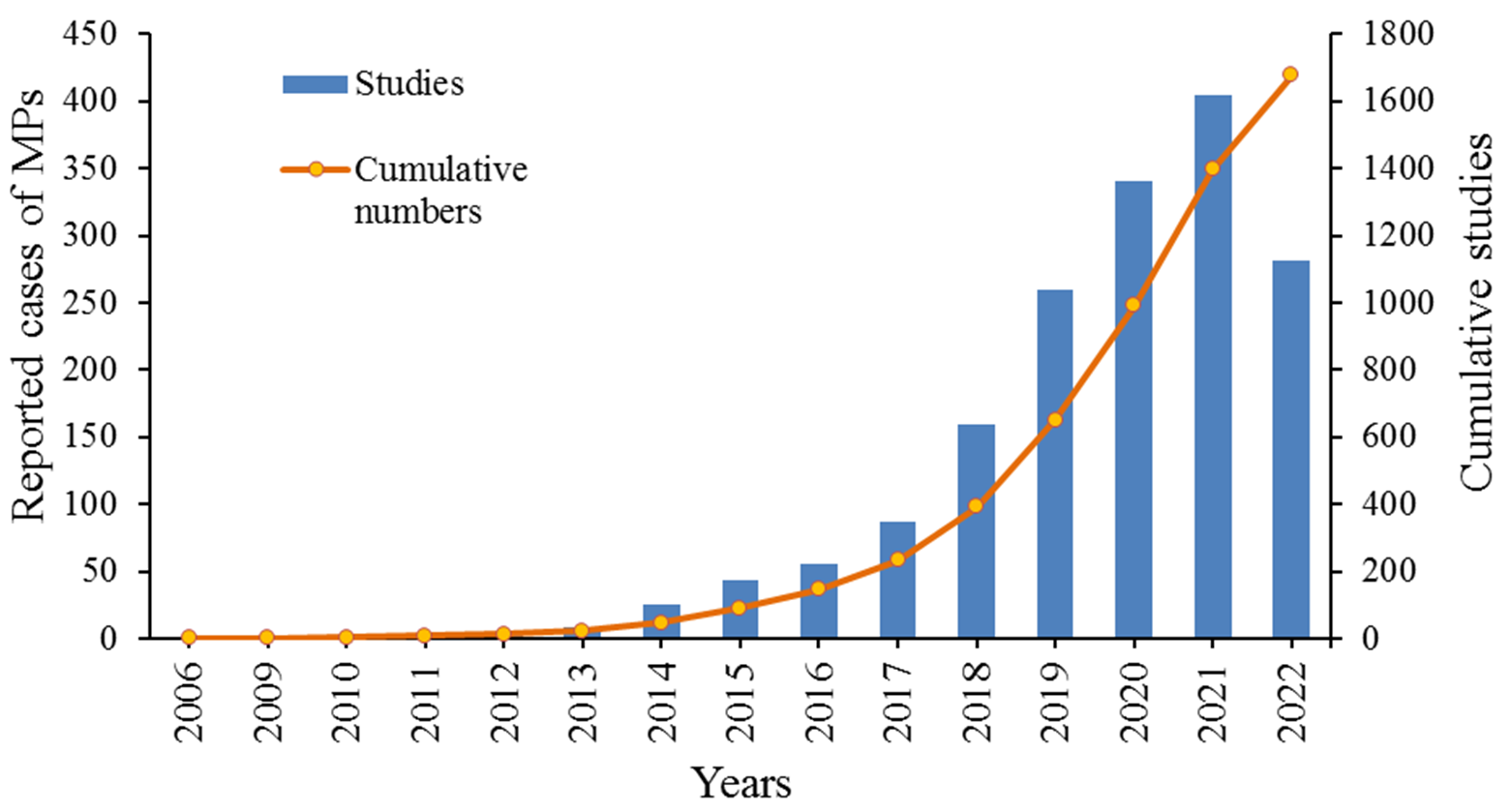


**Fig. S2. Literature growth trend of “MPs/NPs”-related research in aquatic environment**

**Table S1. Recent studies on exposure routes and effects of MPs in different living organisms**

| **Categories** | **Species** | **Plastic types** | **Size** | **Exposure time** | **Exposure media/pathway** | **Reported concentration** | **Exposure routes** | **Effects** | **References** |
| --- | --- | --- | --- | --- | --- | --- | --- | --- | --- |
| Plant | Zea mays | PS-NH_2_ NPs | 22 nm | 28 days | Soil | NR | Primarily, build electrostatic attraction between negatively charged plant cell wall and positively charged PS-NH_2_, then through stomatal openings to vasculature and finally transfer to plant system | Strong inhibitory effect on photosynthesis and higher tendency to activate the cell antioxidant system | Sun et al., 2021 |
|  | Lactuca sativa | PS NPs | 93.6 nm | 30 days | Soil | NR | Absorption of PSNPs through leaves stoma and translocation to root system | Oxidative stress followed by phytotoxicity along with decreased concentration of micronutrients and essential amino acids were observed through foliar exposure of PSNPs in plants | Lian et al., 2021 |
|  | Crocus sativus | PS NPs | 100, 300, 500 and 700 nm | 65 days | Hydroponic | NR | PSNPs entered via piercing the root's epidermal cells. The absorbed PSNPs were assisted in upwards translocation by root pressure and transpiration mechanisms. | Entered PSNPs altered the activity of element transporters in plasma membrane and exchanged the structure of proteins chelating with other elements in the cytoplasm, affected physiological as well as biochemical metabolisms of plant | Li et al., 2021 |
|  | Allium cepa | PS | 50 nm | 3 days | Hydroponic | NR | PSNPs entered by piercing the epidermal tissues and adhering to peripheral root tissues | cytotoxicy, genotoxicity and oxidative damages were reported | Giorgetti et al., 2020 |
|  | *Triticum aestivum*  *Lactuca sativa* | PS and PMMA | 0.2, 2.0, 5.0, 7.0 and 10.0 µm | 10 days  20 days | Hydroponic  Sandy soil | NR | NPs entered through crack-entry channels from lateral root emerging sites and were transferred from root to shoot via transpiration pull mechanisms using vascular system | NR | Li et al., 2020 |
|  | Lepidium sativum | MPs and NPs | 50, 500, and  4800 nm | 3 days | Filter paper | NR | MPs and NPs assembled around the seed capsule, and after rupturing of micropylar endosperm, the accumulated MPs and NPs cling to the emerging radicle. | Suppressed germination because of physical blockage of pores by MPs and NPs in the seed capsule | Bosker et al., 2019 |
| Fish | Silver barb (Barbodes gonionotus) | PVC | 0.1-1000 µm | 4 days | Via water | 0.2-1.0 mg/L | NR | Fish distal and proximal intestine mean thickness increased by 73% and 29%, respectively, in comparison to control. Trypsin and chymotrypsin activities significantly elevated following exposure to PVC MPs. | Romano et al., 2018 |
|  | Zebrafish (Danio rerio) | PS | 0.07-20 µm | 21 days | Via water | 20-2000 µg/L | NR | MPs accumulation found in gills, liver, and gut regions. Increased activities of superoxide dismutase and catalase indicating oxidative stress in cells. Hepatic metabolites changed significantly after MPs exposure in fish | Lu et al., 2016 |
|  | Goldfish (Carassius auratus) | PS NPs | 44 nm | 30 days | Via water | 2.23-266 ng/g liver wet weight  10-19 ng/g of muscle wet weight | NR | PS NPs concentration were higher in liver compared to muscles. Low concentration of PS NPs can damage the DNA by causing the erythrocyte nuclear abnormalities, and cause genotoxicity in fishes | Brandts et al., 2022 |
|  | Gilthead seabream (Sparus aurata) | PMMA-NPs | 45 nm | 1 day and 4 days | Via water | - | NR | PMMA-NPs induced the modulation in lipid pathways genes that increase the cholesterol and triglycerides in plasma. Increased erythrocytic nuclear abnormalities indicates the genotoxicity in fish | Brandts et al., 2021 |
| Microalgae | Scenedesmus obliquus | PS-NPs | 50-100 nm | 4 days | Culture solution | NR | NR | Growth inhibition, disturbance in photosynthesis and cytotoxicity | Liu et al., 2019 |
|  | Chlorella pyrenoidosa | PS | 0.1, 0.3, 1.0 µm | 30 days | Culture solution | NR | NR | Growth inhibition and photosynthesis hindrance | Mao et al., 2018 |
|  | Tetraselmis chuii | MP- procainamide | 1-5 µm | 4 days | Culture solution | NR | NR | Reduced growth and chlorophyll contents | Prata et al., 2018 |

*Note: NR- Not reported*

**Table S2. Methods for identification and quantification of MPs in sediment and water**

| **Environmental samples** | **MPs type** | **Characterization techniques** | | **Advantages** | **Disadvantages** | **References** |
| --- | --- | --- | --- | --- | --- | --- |
|  |  | Visual inspection method | Direct visual method | - Cheap - Non-destructive method of sample processing | - Human-Biasedness - Morphological changes in the weathered MPs make the visual identification even more difficult. | (Crawford and Quinn 2017) |
| Coastal waters | CP, PET, PE |  | Optical microscope observation method | - Provides information on surface texture and structure of MP particles. | - Probability of misidentification by optical techniques increases considerably with decrease in particle size. | (Shim et al. 2017); Liu et al. (2020) |
| Mixed environmental samples (Fish tissues, estuarine system, and marine sediment) | PP, PVC, PE, PET, PS, PU | Thermal analysis method | Pyrolysis gas chromatography mass spectrometry | - MP particles can be directly introduced after minimal sample pre-treatment. - Simultaneously provide detailed information about the chemical composition and organic additives contained in MPs. - Applicable for trace analysis since small amount of sample (100–500 μg) is needed for measurement. | - Only one particle can be analyzed per cycle which requires 30 to 100 min, thus inevitably limiting its applicability for analysis of large sample quantities. - MP particles of size large enough (>100 μm) to be manually manipulated are suitable to be analyzed by the Pyr-GC-MS. | (Käppler et al. 2018); (Nuelle et al. 2014); (Fischer and Scholz-Böttcher 2017); (Gimiliani et al. 2020) |
| Mixed environmental samples (soil, a biogas plant, wastewater treatment plant) | PP, PE, PS |  | Thermal extraction desorption gas chromatography mass spectrometry | - Does not require special sample pre-treatment. - Produces valuable data on polymer mass fraction in environmental samples more rapidly. | - Does not provide direct information on particle sizes and their distribution. | (Dümichen et al. 2017); (Elert et al. 2017) |
| Fresh water Sediment samples | LD-PE, HD-PE, PP |  | Differential scanning colorimetry | - Enables chemical identification and determination of the mass of the particles of a specific polymer. | - Long processing time - Proper sample treatment is required since both identification and mass quantification are influenced by particle size. - Applicable to the identification of only a few types of plastics (PE and PP) due to overlapping thermolytic profiles of plastics. | (Rodríguez Chialanza et al. 2018); (Kurzweg et al. 2024) |
| Marine sediment | PE, PP, PS, PET, PA, PVC | Vibration spectral method | Fourier transform infrared spectroscopy (FTIR) | - Smaller MP particles (>10 μm) can be detected easily by Micro-FTIR. - Direct analysis of large (>500 μm) and irregularly shaped particles without sample preparation using ATR-FTIR. - Provide information about physiochemical weathering of MPs by analyzing their oxidation intensity. | - Has low horizontal resolution and complex spectrums. - Easily gets disturbed by water when detecting wet samples. | (Käppler et al. 2018); (Jaouani et al. 2022) |
| Marine sediment and surface water | PET, HDPE, LDPE, PVC, PP, PC, PA |  | Raman spectroscopy | - Higher spatial resolution and lower sensitivity to water interference. - MPs smaller than 1 μm in size can be identified using a combination of Raman spectroscopy with microscopy (micro-Raman), which is extremely challenging for other spectroscopic techniques to achieve. | - Identification accuracy can be easily interfered with by the presence of additives, pigments or chemicals attached with MPs. | Huppertsberg, and Knepper (2018); (Araujo et al. 2018); (Nava et al. 2021); (Jin et al. 2022) |
|  |  | Auxillary measure method | X-ray photoelectron spectroscopy (XPS) | - Provides information about the surface, small areas, and depth distribution of MPs. | - | (Hu et al. 2020) |
|  |  |  | X-ray diffraction (XRD) | - Provides information on elemental composition of polymers. | - | Turner (2017) |
|  |  |  | Inductive coupled plasma mass spectrometry (ICPMS) | - Detects heavy metal elements in the water, sediments and even on the surface of MPs. | - | (Elseblani et al. 2023) |
|  |  |  | Brunauer-Emmette-Teller | - Reliable analytical technique to measure the surface of MPs. | - | (Moura et al. 2023) |
| Seawater | PE, PP, PS | Other analysis method | Hyperspectral imaging | - Provides images of samples containing hundreds of narrow spectral bands from visible light to the infrared and tens of thousands of pixel space. - Can quickly identify the chemical composition of the MPs and provide information on size, shape, etc. | - Complex operation processes - Low imaging quality. | (Shan et al. 2019); (Ye et al. 2022) |
| Soil samples | PE, PS, PVC |  | Terahertz spectroscopy | - Strong penetration and high sensitivity | - Low spectral signal-to-noise ratio | (Li et al. 2021); (Ye et al. 2022) |
| Marine sediments | PE, PP, PVC, PET, PS |  | Scanning electron microscopy energy dispersive spectroscopy (SEM EDS) | - High resolution images produced by SEM differentiate MPs from other organic or inorganic impurities by examining their surface morphology. - Weathering progress of MPs can be analysed by examining their surface textures (cracks and pits). - Combined use of SEM and EDS provides detailed information about the elemental composition of MPs and the inorganic additives they contain. | - Requires considerable time and effort for sample preparation and thus is not applicable for handling of large number of samples. | (Crawford and Quinn 2017); (Lin et al. 2021) |
| Drinking water | PE, PVC |  | AFM and AFM IR | - Modern technique for identification & characterization of nanoscale MPs. - AFM paired with IR can capture image 50-100 nm spatial resolution, together with specimen’s absorption spectra at same time. | - Finding a nanoscale plastic particle in an unknown material using AFM-IR is hard and takes a lot of time. | (Samanta et al. 2022); (Li et al. 2024) |
|  |  |  | High-performance liquid chromatography (HPLC) | - More suitable for the identification of large, polar, and thermally unstable MPs. - Has high sensitivity and low detection limit; often used as terminal detection method. | - Could be destructive to the sample due to similar chemical composition. | (Fu et al. 2020) |

**References**

Al-Lihaibi, S., Al-Mehmadi, A., Alarif, W.M., Bawakid, N.O., Kallenborn, R. and Ali, A.M., 2019. Microplastics in sediments and fish from the Red Sea coast at Jeddah (Saudi Arabia). Environ. Chem. 16, 641-650. <https://doi.org/10.1071/EN19113>.

Araujo CF, Nolasco MM, Ribeiro AMP, Ribeiro-Claro PJA (2018) Identification of microplastics using Raman spectroscopy: Latest developments and future prospects. Water Research 142:426–440. https://doi.org/10.1016/j.watres.2018.05.060

Barrett, J., Chase, Z., Zhang, J., Holl, M.M.B., Willis, K., Williams, A., Hardesty, B.D., Wilcox, C., 2020. Microplastic pollution in deep-sea sediments from the Great Australian Bight. Front. Mar. Sci. 808. <https://doi.org/10.3389/fmars.2020.576170>.

Bergmann, M., Wirzberger, V., Krumpen, T., Lorenz, C., Primpke, S., Tekman, M.B., Gerdts, G., 2017. High quantities of microplastic in Arctic deep-sea sediments from the HAUSGARTEN observatory. Environ. Sci. Technol. 51, 11000-11010. <https://doi.org/10.1021/acs.est.7b03331>.

Bosker, T., Guaita, L., Behrens, P., 2018. Microplastic pollution on Caribbean beaches in the Lesser Antilles. Mar. Pollut. Bull. 133, 442-447. <https://doi.org/10.1016/j.marpolbul.2018.05.060>.

Chen, L., Yuan, X., Ye, Y., Teng, J., Zhao, J., Wang, Q., Zhang, B., 2022. Characteristics and spatiotemporal distribution of microplastics in sediments from a typical mariculture pond area in Qingduizi Bay, North Yellow Sea, China. Mar. Pollut. Bull. 176, 113436. <https://doi.org/10.1016/j.marpolbul.2022.113436>.

Chubarenko, I., Esiukova, E., Zobkov, M., Isachenko, I., 2022. Microplastics distribution in bottom sediments of the Baltic Sea Proper. Mar. Pollut. Bull. 179, 113743. <https://doi.org/10.1016/j.marpolbul.2022.113743>.

Cincinelli, A., Scopetani, C., Chelazzi, D., Martellini, T., Pogojeva, M., Slobodnik, J., 2021. Microplastics in the Black Sea sediments. Sci. Total Environ. 760, 143898. <https://doi.org/10.1016/j.scitotenv.2020.143898>.

Claessens, M., De Meester, S., Van Landuyt, L., De Clerck, K., Janssen, C.R., 2011. Occurrence and distribution of microplastics in marine sediments along the Belgian coast. Mar. Pollut. Bull. 62, 2199-2204. <https://doi.org/10.1016/j.marpolbul.2011.06.030>.

Crawford CB, Quinn B (2017) Microplastic pollutants. Elsevier, Amsterdam

Cui, Y., Liu, M., Selvam, S., Ding, Y., Wu, Q., Pitchaimani, V.S., Huang, P., Ke, H., Zheng, H., Liu, F., Luo, B., 2022. Microplastics in the surface waters of the South China sea and the western Pacific Ocean: Different size classes reflecting various sources and transport. Chemosphere 299, 134456. <https://doi.org/10.1016/j.chemosphere.2022.134456>.

De Villiers, S., 2018. Quantification of microfibre levels in South Africa's beach sediments, and evaluation of spatial and temporal variability from 2016 to 2017. Mar. Pollut. Bull. 135, 481-489. <https://doi.org/10.1016/j.marpolbul.2018.07.058>.

Dümichen E, Eisentraut P, Bannick CG, et al (2017) Fast identification of microplastics in complex environmental samples by a thermal degradation method. Chemosphere 174:572–584. https://doi.org/10.1016/j.chemosphere.2017.02.010

Elert AM, Becker R, Duemichen E, et al (2017) Comparison of different methods for MP detection: What can we learn from them, and why asking the right question before measurements matters? Environmental Pollution 231:1256–1264. https://doi.org/10.1016/j.envpol.2017.08.074

Eryaşar, A.R., Gedik, K., Şahin, A., Öztürk, R.Ç., Yılmaz, F., 2021. Characteristics and temporal trends of microplastics in the coastal area in the Southern Black Sea over the past decade. Mar. Pollut. Bull. 173, 112993. <https://doi.org/10.1016/j.marpolbul.2021.112993>.

Expósito, N., Rovira, J., Sierra, J., Folch, J, Schuhmacher, M., 2021. Microplastics levels, size, morphology and composition in marine water, sediments and sand beaches. Case study of Tarragona coast (western Mediterranean). Sci. Total Environ., 786, 147453. <https://doi.org/10.1016/j.scitotenv.2021.147453>.

Fischer M, Scholz-Böttcher BM (2017) Simultaneous Trace Identification and Quantification of Common Types of Microplastics in Environmental Samples by Pyrolysis-Gas Chromatography–Mass Spectrometry. Environ Sci Technol 51:5052–5060. https://doi.org/10.1021/acs.est.6b06362

Fu W, Min J, Jiang W, et al (2020) Separation, characterization and identification of microplastics and nanoplastics in the environment. Science of The Total Environment 721:137561. https://doi.org/10.1016/j.scitotenv.2020.137561

Gimiliani GT, Fornari M, Redígolo MM, et al (2020) Simple and cost-effective method for microplastic quantification in estuarine sediment: A case study of the Santos and São Vicente Estuarine System. Case Studies in Chemical and Environmental Engineering 2:100020. https://doi.org/10.1016/j.cscee.2020.100020

Goswami, P., Vinithkumar, N.V., Dharani, G., 2020. First evidence of microplastics bioaccumulation by marine organisms in the Port Blair Bay, Andaman Islands. Mar. Pollut. Bull. 155, 111163. <https://doi.org/10.1016/j.marpolbul.2020.111163>.

Goswami, P., Vinithkumar, N.V., Dharani, G., 2021. Microplastics particles in seafloor sediments along the Arabian Sea and the Andaman Sea continental shelves: First insight on the occurrence, identification, and characterization. Mar. Pollut. Bull. 167, 112311. <https://doi.org/10.1016/j.marpolbul.2021.112311>.

Hu B, Li Y, Jiang L, et al (2020) Influence of microplastics occurrence on the adsorption of 17β-estradiol in soil. Journal of Hazardous Materials 400:123325. https://doi.org/10.1016/j.jhazmat.2020.123325

Jaouani R, Mouneyrac C, Châtel A, et al (2022) Seasonal and spatial distribution of microplastics in sediments by FTIR imaging throughout a continuum lake - lagoon- beach from the Tunisian coast. Science of The Total Environment 838:156519. https://doi.org/10.1016/j.scitotenv.2022.156519

Jin N, Song Y, Ma R, et al (2022) Characterization and identification of microplastics using Raman spectroscopy coupled with multivariate analysis. Analytica Chimica Acta 1197:339519. https://doi.org/10.1016/j.aca.2022.339519

Kurzweg L, Hauffe M, Schirrmeister S, et al (2024) Microplastic analysis in sediments of the Elbe River by electrostatic separation and differential scanning calorimetry. Science of The Total Environment 930:172514. https://doi.org/10.1016/j.scitotenv.2024.172514

Käppler A, Fischer M, Scholz-Böttcher BM, et al (2018) Comparison of μ-ATR-FTIR spectroscopy and py-GCMS as identification tools for microplastic particles and fibers isolated from river sediments. Anal Bioanal Chem 410:5313–5327. https://doi.org/10.1007/s00216-018-1185-5

Li Y, Yao J, Nie P, et al (2021) An effective method for the rapid detection of microplastics in soil. Chemosphere 276:128696. https://doi.org/10.1016/j.chemosphere.2020.128696

Li Y, Zhang C, Tian Z, et al (2024) Identification and quantification of nanoplastics (20–1000 nm) in a drinking water treatment plant using AFM-IR and Pyr-GC/MS. Journal of Hazardous Materials 463:132933. https://doi.org/10.1016/j.jhazmat.2023.132933

Lin J, Xu X-P, Yue B-Y, et al (2021) A novel thermoanalytical method for quantifying microplastics in marine sediments. Science of The Total Environment 760:144316. https://doi.org/10.1016/j.scitotenv.2020.144316

Lorenz, C., Roscher, L., Meyer, M.S., Hildebrandt, L., Prume, J., Löder, M.G., Primpke, S., Gerdts, G., 2019. Spatial distribution of microplastics in sediments and surface waters of the southern North Sea. Environ. Poll. 252, 1719-1729. <https://doi.org/10.1016/j.envpol.2019.06.093>.

Manbohi, A., Mehdinia, A., Rahnama, R., Dehbandi, R., 2021a. Microplastic pollution in inshore and offshore surface waters of the southern Caspian Sea. Chemosphere 281, 130896. <https://doi.org/10.1016/j.chemosphere.2021.130896>.

Manbohi, A., Mehdinia, A., Rahnama, R., Dehbandi, R., Hamzehpour, A., 2021b. Spatial distribution of microplastics in sandy beach and inshore-offshore sediments of the southern Caspian Sea. Mar. Pollut. Bull. 169, 112578. <https://doi.org/10.1016/j.marpolbul.2021.112578>.

Matsuguma, Y., Takada, H., Kumata, H., Kanke, H., Sakurai, S., Suzuki, T., Itoh, M., Okazaki, Y., Boonyatumanond, R., Zakaria, M.P., Weerts, S., 2017. Microplastics in sediment cores from Asia and Africa as indicators of temporal trends in plastic pollution. Arch. Environ. Contam. Toxicol. 73, 230-239. <https://doi.org/10.1007/s00244-017-0414-9>.

Mishra, A., Buhhalko, N., Lind, K., Lips, I., Liblik, T., Väli, G., Lips, U., 2022. Spatiotemporal Variability of Microplastics in the Eastern Baltic Sea. Front. Mar. Sci. 686. <https://doi.org/10.3389/fmars.2022.875984>.

Mistri, M., Scoponi, M., Granata, T., Moruzzi, L., Massara, F., Munari, C., 2020. Types, occurrence and distribution of microplastics in sediments from the northern Tyrrhenian Sea. Mar. Pollut. Bull. 153, 111016. <https://doi.org/10.1016/j.marpolbul.2020.111016>.

Moura DS, Pestana CJ, Moffat CF, et al (2023) Characterisation of microplastics is key for reliable data interpretation. Chemosphere 331:138691. https://doi.org/10.1016/j.chemosphere.2023.138691

Mu, J., Qu, L., Jin, F., Zhang, S., Fang, C., Ma, X., Zhang, W., Huo, C., Cong, Y., Wang, J., 2019. Abundance and distribution of microplastics in the surface sediments from the northern Bering and Chukchi Seas. Environ. Poll. 245, 122-130. <https://doi.org/10.1016/j.envpol.2018.10.097>.

Nava V, Frezzotti ML, Leoni B (2021) Raman Spectroscopy for the Analysis of Microplastics in Aquatic Systems. Appl Spectrosc 75:1341–1357. https://doi.org/10.1177/00037028211043119

Nguyen, Q.A.T., Nguyen, H.N.Y., Strady, E., Nguyen, Q.T., Trinh-Dang, M., 2020. Characteristics of microplastics in shoreline sediments from a tropical and urbanized beach (Da Nang, Vietnam). Mar. Pollut. Bull. 161, 111768. <https://doi.org/10.1016/j.marpolbul.2020.111768>.

Nuelle M-T, Dekiff JH, Remy D, Fries E (2014) A new analytical approach for monitoring microplastics in marine sediments. Environmental Pollution 184:161–169. https://doi.org/10.1016/j.envpol.2013.07.027

Pojar, I., Stănică, A., Stock, F., Kochleus, C., Schultz, M., Bradley, C., 2021. Sedimentary microplastic concentrations from the Romanian Danube River to the Black Sea. Sci. Rep. 11, pp.1-9. <https://doi.org/10.1038/s41598-021-81724-4>.

Rangel-Buitrago, N., Arroyo-Olarte, H., Trilleras, J., Arana, V.A., Mantilla-Barbosa, E., Gracia, A., Mendoza, A.V., Neal, W.J., Williams, A.T., Micallef, A., 2021. Microplastics pollution on colombian Central Caribbean beaches. Mar. Pollut. Bull. 170, 112685. <https://doi.org/10.1016/j.marpolbul.2021.112685>.

Ranjani, M., Veerasingam, S., Venkatachalapathy, R., Mugilarasan, M., Bagaev, A., Mukhanov, V., Vethamony, P.J.M.P.B., 2021. Assessment of potential ecological risk of microplastics in the coastal sediments of India: A meta-analysis. Mar. Pollut. Bull 163, 111969. <https://doi.org/10.1016/j.marpolbul.2021.111969>.

Rodríguez Chialanza M, Sierra I, Pérez Parada A, Fornaro L (2018) Identification and quantitation of semi-crystalline microplastics using image analysis and differential scanning calorimetry. Environ Sci Pollut Res 25:16767–16775. https://doi.org/10.1007/s11356-018-1846-0

Ruiz-Compean, P., Ellis, J., Cúrdia, J., Payumo, R., Langner, U., Jones, B., Carvalho, S., 2017. Baseline evaluation of sediment contamination in the shallow coastal areas of Saudi Arabian Red Sea. Mar. Pollut. Bull. 123, 205-218. <https://doi.org/10.1016/j.marpolbul.2017.08.059>.

Samanta P, Dey S, Kundu D, et al (2022) An insight on sampling, identification, quantification and characteristics of microplastics in solid wastes. Trends in Environmental Analytical Chemistry 36:e00181. https://doi.org/10.1016/j.teac.2022.e00181

Shan J, Zhao J, Zhang Y, et al (2019) Simple and rapid detection of microplastics in seawater using hyperspectral imaging technology. Analytica Chimica Acta 1050:161–168. https://doi.org/10.1016/j.aca.2018.11.008

Shim WJ, Hong SH, Eo SE (2017) Identification methods in microplastic analysis: a review. Anal Methods 9:1384–1391. https://doi.org/10.1039/C6AY02558G

Urban-Malinga, B., Zalewski, M., Jakubowska, A., Wodzinowski, T., Malinga, M., Pałys, B., Dąbrowska, A., 2020. Microplastics on sandy beaches of the southern Baltic Sea. Mar. Pollut. Bull. 155, 111170. <https://doi.org/10.1016/j.marpolbul.2020.111170>.

Yaranal, N.A., Subbiah, S., Mohanty, K., 2021. Distribution and characterization of microplastics in beach sediments from Karnataka (India) coastal environments. Mar. Pollut. Bull. 169, 112550. <https://doi.org/10.1016/j.marpolbul.2021.112550>.

Ye Y, Yu K, Zhao Y (2022) The development and application of advanced analytical methods in microplastics contamination detection: A critical review. Science of The Total Environment 818:151851. https://doi.org/10.1016/j.scitotenv.2021.151851

Yu, X., Peng, J., Wang, J., Wang, K., Bao, S., 2016. Occurrence of microplastics in the beach sand of the Chinese inner sea: the Bohai Sea. Environ. Poll. 214, 722-730. <https://doi.org/10.1016/j.envpol.2016.04.080>.

Zhang, M., Lin, Y., Booth, A.M., Song, X., Cui, Y., Xia, B., Gu, Z., Li, Y., Liu, F., Cai, M., 2022. Fate, source and mass budget of sedimentary microplastics in the Bohai Sea and the Yellow Sea. Environ. Poll. 294, 118640. <https://doi.org/10.1016/j.envpol.2021.118640>.

Zobkov, M., Esiukova, E., 2017. Microplastics in Baltic bottom sediments: quantification procedures and first results. Mar. Pollut. Bull. 114, 724-732. <https://doi.org/10.1016/j.marpolbul.2016.10.060>.
